# Neural Computations Mediating One-Shot Learning in the Human Brain

**DOI:** 10.1371/journal.pbio.1002137

**Published:** 2015-04-28

**Authors:** Sang Wan Lee, John P. O’Doherty, Shinsuke Shimojo

**Affiliations:** 1 Computation & Neural Systems, MC228-77, California Institute of Technology, Pasadena, California, United States of America; 2 Behavioral & Social Neuroscience, MC228-77, California Institute of Technology, Pasadena, California, United States of America; 3 Division of Humanities and Social Sciences, MC228-77, California Institute of Technology, Pasadena, California, United States of America; 4 Division of Biology and Biological Engineering, MC228-77, California Institute of Technology, Pasadena, California, United States of America; University of Oxford, UNITED KINGDOM

## Abstract

Incremental learning, in which new knowledge is acquired gradually through trial and error, can be distinguished from one-shot learning, in which the brain learns rapidly from only a single pairing of a stimulus and a consequence. Very little is known about how the brain transitions between these two fundamentally different forms of learning. Here we test a computational hypothesis that uncertainty about the causal relationship between a stimulus and an outcome induces rapid changes in the rate of learning, which in turn mediates the transition between incremental and one-shot learning. By using a novel behavioral task in combination with functional magnetic resonance imaging (fMRI) data from human volunteers, we found evidence implicating the ventrolateral prefrontal cortex and hippocampus in this process. The hippocampus was selectively “switched” on when one-shot learning was predicted to occur, while the ventrolateral prefrontal cortex was found to encode uncertainty about the causal association, exhibiting increased coupling with the hippocampus for high-learning rates, suggesting this region may act as a “switch,” turning on and off one-shot learning as required.

## Abstract 2

A combination of neuroimaging and computational modeling suggests that a part of ventrolateral prefrontal cortex, in cooperation with the hippocampus, is responsible for switching between incremental and one-shot strategies for learning about causal relationships. Read the Synopsis.

## Author Summary

There are at least two distinct learning strategies for identifying the relationship between a cause and its consequence: (1) incremental learning, in which we gradually acquire knowledge through trial and error, and (2) one-shot learning, in which we rapidly learn from only a single pairing of a potential cause and a consequence. Little is known about how the brain switches between these two forms of learning. In this study, we provide evidence that the amount of uncertainty about the relationship between cause and consequence mediates the transition between incremental and one-shot learning. Specifically, the more uncertainty there is about the causal relationship, the higher the learning rate that is assigned to that stimulus. By imaging the brain while participants were performing the learning task, we also found that uncertainty about the causal association is encoded in the ventrolateral prefrontal cortex and that the degree of coupling between this region and the hippocampus increases during one-shot learning. We speculate that this prefrontal region may act as a “switch,” turning on and off one-shot learning as required.

## Introduction

In standard associative learning, an animal must repeatedly experience a number of pairings between a stimulus and a consequence before a particular stimulus pairing is fully learned. Learning is inevitably incremental. However, animals sometimes encounter outcomes that they have never experienced previously and from which it is necessary to learn rapidly in order to survive. In such cases, animals can learn on the basis of only a single exposure to a stimulus pairing, a situation described in the literature as one-shot learning. For example, in one-shot object categorization, humans demonstrate a capability to rapidly learn to recognize novel objects by means of a priori knowledge of object categories [1]. In jumping to conclusions, humans are known to undergo a rapid inference process because of an overestimation of the cost of acquiring more information [2–4]. In (mis)attribution, an outcome can be (erroneously) attributed to a (wrong) cause [4,5]. Owing to these findings, we have made progress in elucidating distinctive traits of one-shot learning at the behavioral level. However, we still have only a rudimentary understanding of the computational principles underpinning one-shot learning, and therefore, how this computational process unfolds at the neural level remains largely unknown.

Much progress has been made in understanding the computational mechanisms underpinning incremental learning with algorithms such as the Rescorla-Wagner rule [6], the probabilistic contrast model [7], the associative learning model [8,9], and Bayesian causal inference [4,10–12], providing computational accounts for a wide variety of types of incremental learning. In these models, repeated experience of the same stimulus and outcome gradually cements the causal relationship between them until there is little left to learn. However, one-shot learning imposes a substantial challenge to these learning algorithms because such models are not optimized to facilitate learning from a single experience. One viable mechanism for switching between incremental and one-shot learning might be via the control of learning rates. It has been suggested in previous theoretical proposals that learning rate is modulated through changes in environmental uncertainty, such as volatility [8,13,14]. However, these prior proposals are not designed to account for one-shot learning because learning rates are adjusted only gradually in such frameworks based on detecting changes in environmental volatility or jumps in contingencies [13,14].

One process that is likely to contribute substantially to one-shot learning is episodic memory [15,16], in which rapid associations between a context and an event can be formed [17–22]. This type of memory has long been known to depend at least in part on the hippocampal complex [23–27], which has also been proposed to be both functionally and anatomically dissociable from other forms of memory involved in mediating more incremental types of associative learning [15,20,28,29]. However, while much evidence has accumulated in support of the notion of dissociable memory systems for one-shot and incremental learning [15,28,30–33], almost nothing is known about how the brain is capable of switching between different types of learning strategy. In other words, how does the brain know when to deploy the episodic memory system as opposed to relying on incremental learning?

The aim of the present study is to test a novel computational framework that can account for when and how one-shot learning occurs over and above incremental learning, as well as to gain insight into how the brain is capable of implementing the switch between these different learning strategies. Our computational hypothesis is that the rates at which individuals learn to causally associate a stimulus with an outcome increases with the extent to which the relative amount of uncertainty in the causal relationship between that stimulus and outcome are left unresolved. Specifically, the more uncertainty there is about the causal relationship between a stimulus and an outcome, the higher the learning rate that is assigned to that stimulus in order to resolve the uncertainty. Stimulus-outcome pairs with very high uncertainty associated with them should elicit very rapid one-shot learning.

At the neural level, the ventrolateral prefrontal cortex (vlPFC) has long been hypothesized to guide a control process that determines whether items are remembered or forgotten during episodic encoding [34–40]. It has further been established that interplay between the vlPFC and the hippocampus increases more when learning stronger associations than when learning weaker ones [36] and that these structures exhibit elevated connectivity during demanding tasks relative to less demanding ones [41]. Considering the functional similarity between episodic memory processes and one-shot learning, we hypothesized that areas involved in episodic memory processing such as the hippocampus might be selectively engaged under situations in which one-shot learning occurs. We further hypothesized that recruitment of the hippocampus might depend on uncertainty computations about the causal relationship mediated in parts of the prefrontal cortex—particularly the lateral prefrontal cortex, which has been implicated in human causal learning in previous studies [42–45].

## Results

### Causal Learning Task

**Fig 1:**
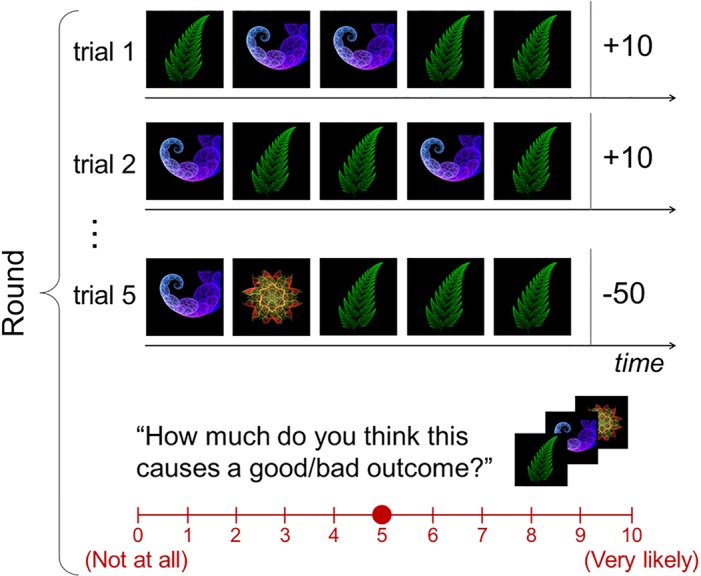
Causal learning task. Task design. On each trial, participants are presented with a sequence of pictures that vary in the degree of frequency in which they are presented, followed by receiving one of two types of monetary outcome—non-novel and novel outcomes. Each picture and outcome is displayed for 1 and 2 s, respectively. The presentations of successive pictures and individual trials are separated by a variable temporal interval drawn from a uniform distribution between 1 to 4 s. After the fifth trial, they are asked to make ratings about each stimulus-outcome pair in turn. A maximum of 4 s is allowed for each submission. Participants were asked to complete 40 rounds.

To test our hypotheses, we combined formal computational models with behavioral and neuroimaging (functional magnetic resonance imaging [fMRI]) data acquired from individuals performing a simple causal inference task, in which learning can occur from a single experience. Measuring neural signals for one-shot learning is challenging because by virtue of how rapidly it happens, there is very little time to collect data samples while it is going on in the brain. To resolve this issue, we developed a novel paradigm that enables us to assess one-shot learning (Fig 1). On each trial, participants are presented with a sequence of pictures. These pictures vary in the degree of frequency in which they are presented (“novel cue” for the least frequently presented pictures and “non-novel cue” otherwise). After the sequence of pictures has been presented, participants will receive a monetary outcome, for which neither response nor choice is needed. This outcome will in some cases be to win a certain amount of money and on other occasions will involve losing a certain amount of money (see Materials and Methods for more details). One type of outcome is presented frequently (“non-novel outcome”), while the other type is presented only once (“novel outcome”). After all the outcomes are presented in a round, participants are then asked to make ratings about how likely it is that each individual stimulus encountered during the round can cause an outcome (“causal rating”). Participants were told that all rounds are independent of each other, and no participant reported noticing any dependencies between rounds while performing the task.

Passive viewing of these sequential stimuli and outcome presentations allows us to test a pure effect of learning, without the confounding effects of choice behavior. Another important feature of this task is that the stimuli and outcomes do not necessarily occur contiguously—that is, multiple different stimuli are presented before each outcome is presented. This makes the present paradigm quite different from typical associative learning paradigms such as Pavlovian conditioning, in which a single cue is usually followed by an outcome (or the absence of an outcome).

The design of our task was structured so as to enable us to distinguish between incremental and one-shot learning. The task is designed, as will be described below, to enable us to test whether one-shot learning occurs when the amount of uncertainty in the causal relationship between a stimulus and an outcome is greater than that of other stimulus-outcome pairs.

### Computational Model for Causal Learning

**Fig 2:**
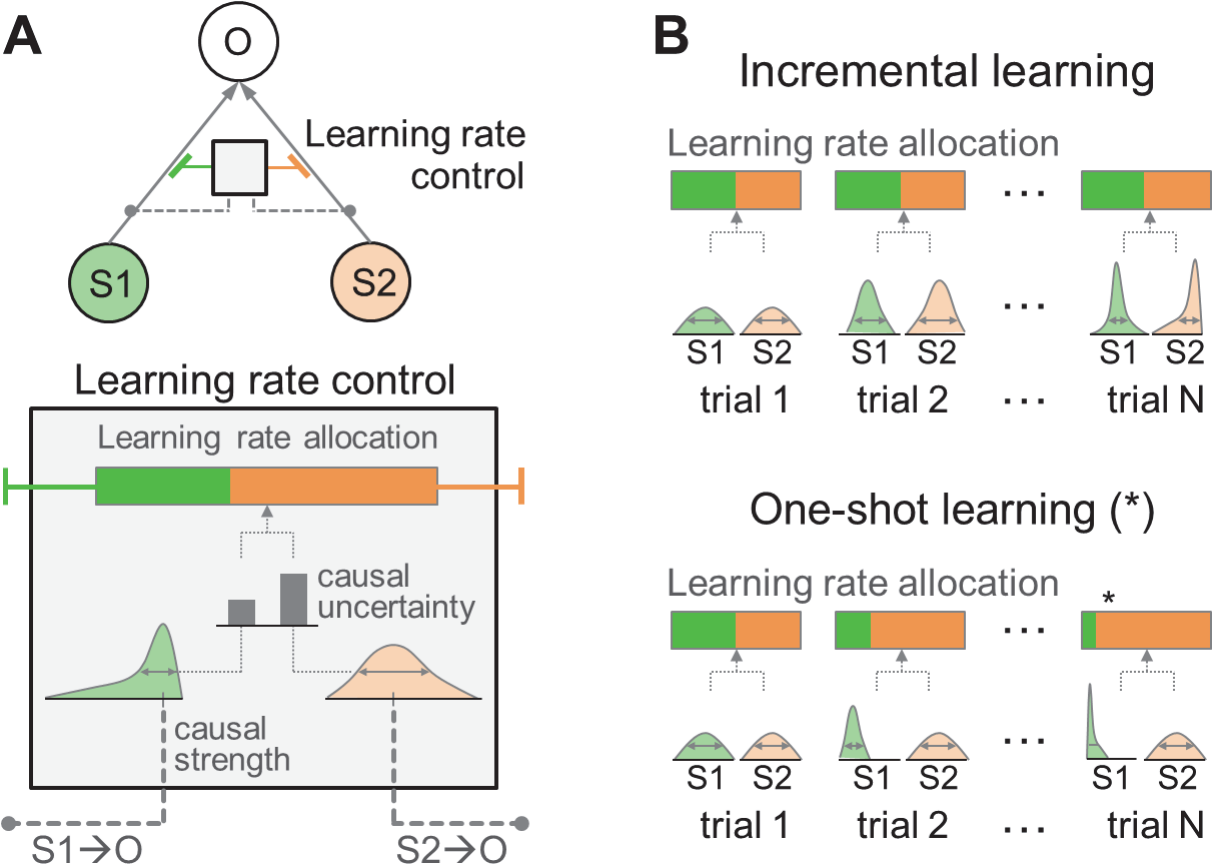
Causal learning model. (A) Causal uncertainty model. The causal relationship between a stimulus (S1 or S2) and an outcome (O) can be encapsulated as a probability distribution, of which a mean and a variance are referred to as a “causal strength” and a “causal uncertainty,” respectively. The grey arrow indicates the causal relationship, and color indicates a category of causal relationship (green for the S1-O pair and orange for the S2-O pair). We hypothesized that the role of causal uncertainty is pivotal in determining the rate at which new information is taken into account to update one’s current predictions about the causal relationship. The learning rate for a particular stimulus is dictated by the process of determining how much causal uncertainty is greater for a given stimulus than for other stimuli in the environment (“learning rate allocation”). (B) Learning rate control of the causal learning model. Incremental learning: in a situation in which the causal uncertainty for both the S1-O and the S2-O is gradually resolved over the course of trials—such as, for example, when S2 is a probable cause of the O and the S1 is not—the variance of each probability distribution would decrease at an almost equal rate. Accordingly, both S1-O and S2-O would be assigned equal learning rates. One-shot learning: if the causal uncertainty for the S1-O is quickly resolved while the causal uncertainty for the S2-O remains unresolved, then the variance of the probability distribution for the S1-O pair would be smaller than that for the S2-O pair. It will lead to a situation in which the learning rate applied to the S2-O pair becomes much greater than that for the S1-O pair. This proactive strategy for causal learning allows the model to allocate learning rates in a way that rapidly reduces the amount of causal uncertainty left unresolved in the previous trial.

The computational model we propose to account for one-shot learning is based on a probabilistic instantiation of a causal learning model (Fig 2). It builds on the premise that one-shot learning is characterized by a dramatic increase of learning rate (the rate at which new information is taken into account to update one’s current predictions). Such an increase in learning rate is hypothesized to occur when uncertainty in the causal relationship between a stimulus and an outcome is maximal, with the rate decreasing as the uncertainty is resolved. To implement this hypothesis, we constructed a normative Bayesian model for one-shot learning in which a Bayesian learner attempts to establish the causal relationship between a stimulus and an outcome (“causal strength” [10,11]) and the uncertainty about the causal strength (“causal uncertainty”), whilst the degree of relative uncertainty about different possible causal relationships modulates the learning rate.

Specifically, the model performs a probability estimate about the extent to which a particular stimulus has caused a given outcome (see Fig 2A and Materials and Methods, section “Bayesian inference for causal learning,” for more details). This is specified by the parameters of the posterior distribution, which is updated at the end of each trial when an outcome is presented. The mean and the variance of the posterior are referred to as the causal strength and the causal uncertainty, respectively. We speculate that the former is an observable variable reflected in actual rating patterns and the latter is a latent variable reflected in neural patterns that essentially weave those rating patterns.

The amount of causal uncertainty for each stimulus and outcome pair is then compared and translated into the learning rate by means of the softmax function (see Fig 2B) [46]. This process of learning rate control assumes that the rate of learning to resolve remaining causal uncertainty about the stimulus-outcome pair at present increases with the amount of causal uncertainty left unresolved for this pair, compared to the amount of causal uncertainty for other pairs. For example, if causal uncertainties about all possible stimulus-outcome pairings were almost equal to each other, the brain would allocate learning rates evenly to all pairs, resulting in slow learning (refer to “incremental learning” in Fig 2B). Conversely, a stimulus paired with an outcome in which the amount of causal uncertainty is significantly greater than other possible stimulus-outcome pairings results in very rapid learning, such that even within a single trial substantial learning occurs (refer to “one-shot learning” in Fig 2B). Note that the model updates its posterior distributions about the stimulus-outcome causal relationships on a trial-by-trial basis (when an outcome is presented) while assigning learning rates to each individual stimulus-outcome pair on an event-by-event basis (when each stimulus is presented). An assessment of the model’s viability and supporting simulation results demonstrating that the learning rate assignment effectively reduces the amount of causal uncertainty are provided in Materials and Methods and S1 Fig and S2 Fig.

It is noted that our model reduces to a simpler heuristic model if we assume that causal uncertainty is high whenever the stimulus and the outcome novelty are high. This assumption leads us to consider an alternative hypothesis stating that the causal learning process is purely driven by the novelty of the stimulus-outcome pair. This can also be viewed as reflecting the operation of a simple heuristic causal judgment that a novel stimulus is rated as the most probable cause of a novel outcome if the novel stimulus is paired with that novel outcome.

The event-by-event distinction between incremental and one-shot learning is made by simply reading out the learning rate for the stimulus presented at the time of each event. High learning rates at the time of stimulus event, reflecting the extent to which the relative amount of uncertainty in the causal relationship between that stimulus and outcome are left unresolved, imply that the model has high expectations that the stimulus event will be followed by an “informative” outcome event, by which time causal uncertainty is likely to be resolved. The one-shot learning we refer to here is thus an expectation or preparedness for an event that enables a decrease in the remaining amount of causal uncertainty, regardless of whether or not a novel outcome is presented at the end of the current trial. This allows us not only to test predictions of our causal uncertainty model above and beyond the distinction drawn by the novelty of the stimulus-outcome pair but also to dissociate neural processes pertaining to one-shot learning from those involved in incremental learning.

### Behavioral Results

Forty-seven adult participants (14 females, between the ages of 19 and 43, mean = 25.8, standard deviation = 5.2) performed the task in total. Twenty (ten females, between the ages of 19 and 40, mean = 26.1, standard deviation = 5.3) among them were scanned with fMRI, and another 27 (four females, between the ages of 20 and 43, mean = 25.6, standard deviation = 5.3) performed the task in follow-up behavioral experiments.

One generic characteristic of our causal uncertainty model is that uncertainty will generally be high when a novel stimulus is paired with a novel outcome. To test for this effect, we created two round categories by stratifying rounds by the novelty of the stimulus-outcome pair. In the type1 rounds, a novel cue is paired with a non-novel outcome. In the type2 rounds, a novel cue is paired with a novel outcome. In both cases, non-novel cues are paired with both the novel and the non-novel outcomes.

**Fig 3:**
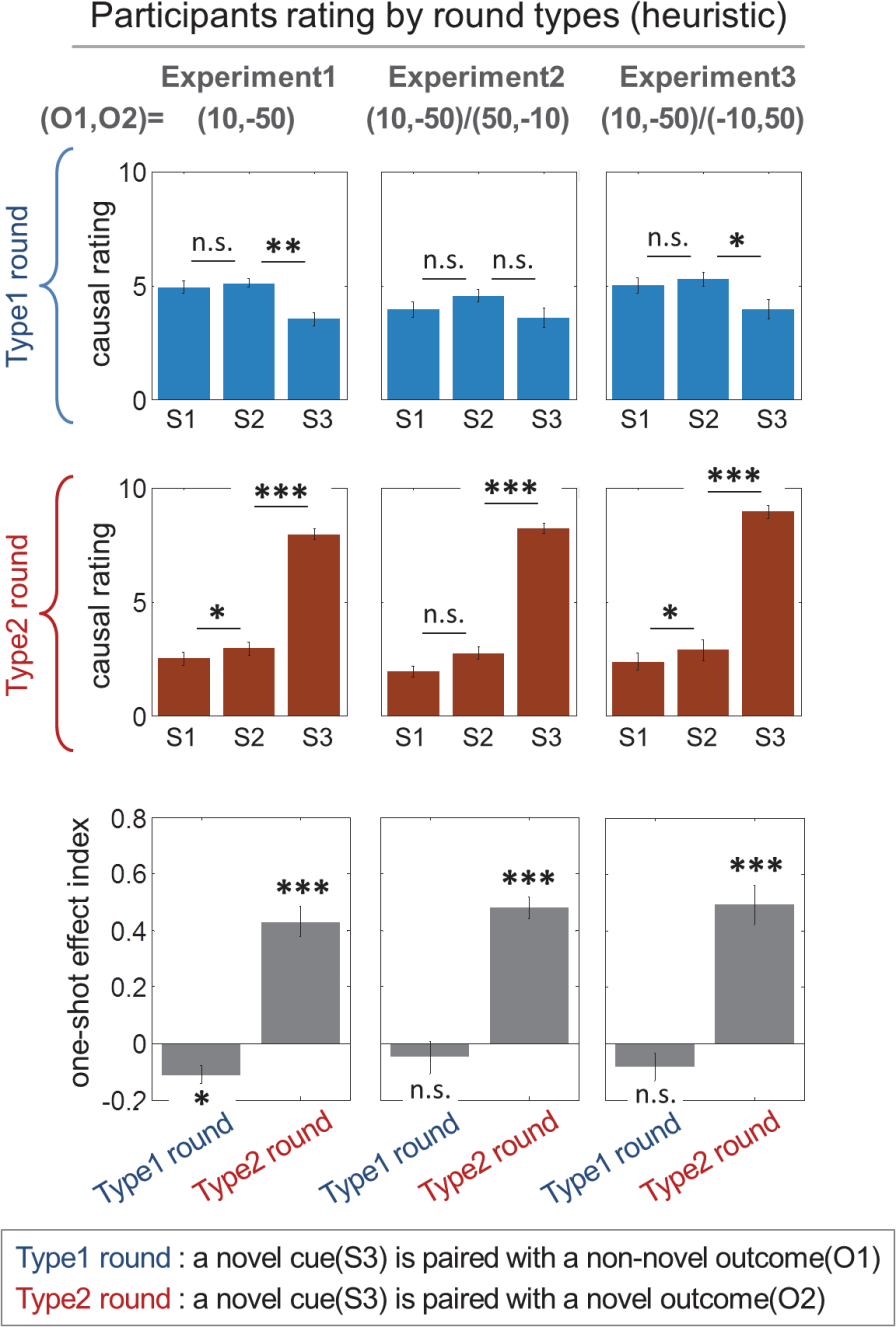
Behavioral results. Participants rating patterns classified according to stimulus-outcome novelty pairings (heuristic causal judgment). O1,O2 indicates an outcome condition, where O1 refers to the amount of the non-novel outcome and O2 refers to the amount of the novel outcome. The type1 round and the type2 round refer to rounds in which a novel cue is paired with a non-novel outcome and with a novel outcome, respectively. S1 and S2 refer to the non-novel cues, and S3 refers to a novel cue that is presented only once each round. The first two rows (“causal rating”) show subjects’ causal ratings (scale: 0–10) describing the subjective judgment about the extent to which a given stimulus caused the novel outcome on each trial type as a function of stimulus novelty. Each column refers to the experiment in which different magnitudes of outcomes are used, as indicated by O1,O2. In the first experiment, participants performed a task while being scanned with fMRI. The second and the third experiment were follow-up behavioral experiments (see Materials and Methods; test statistics are provided in the main text). The third row (“one-shot effect index”) illustrates the quantification of the one-shot learning effect in the causal rating dataset. The one-shot effect index is defined as the causal rating for the novel cue minus the average causal ratings for the non-novel cues. The one-shot learning effect indices are significantly positive in the type2 rounds, whereas they are zero or negative in the type1 rounds. *: *p* < 1e-2, **: *p* < 1e-3, ***: *p* < 1e-4; paired-sample *t* test for underlined asterisks and one-sample *t* test for asterisks without an underline. Error bars are standard error of the mean (SEM) across subjects.

As expected, we found that a majority of participants rated the novel stimulus as the most probable cause of the novel outcome in the type2 round, as opposed to the type1 round (Fig 3; *p* < 1e-4, paired-sample *t* test with the causal rating dataset and one sample *t* test with the one-shot effect index). This one-shot learning effect occurs regardless of any delay between the novel stimulus and the novel outcome within a trial; we found no significant correlation between the distance between a novel stimulus and an outcome and the one-shot effect, which is defined as the causal rating for the novel cue minus the average causal ratings for the non-novel cues (correlation coefficients = -0.002, *p* = 0.95). This implies that the model successfully predicts the effect of stimulus-outcome novelty.

In two follow-up behavioral experiments, we examined if there is an effect of the sign of the outcome (whether the novel outcome is a gain or a loss) and of the magnitude of the outcome on the causal ratings (see Materials and Methods for more details). While we found no effect of sign (Fig 3—Experiment3; two-way repeated-measure ANOVA; F-test = 0.52, *p* = 0.46), a modest effect of outcome magnitude was found on the extent to which one-shot learning occurs when systematically examined (Fig 3—Experiment2; two-way repeated-measure ANOVA; F-test = 4.31, *p* = 0.04), suggesting that participants do take the amount of outcome into account when making causality judgments. In both of the behavioral follow-up experiments, the one-shot learning effect was observed (two-way repeated-measure ANOVA; F-test > 200, *p* < 1e-20).

### Beyond Stimulus-Outcome Novelty

**Fig 4:**
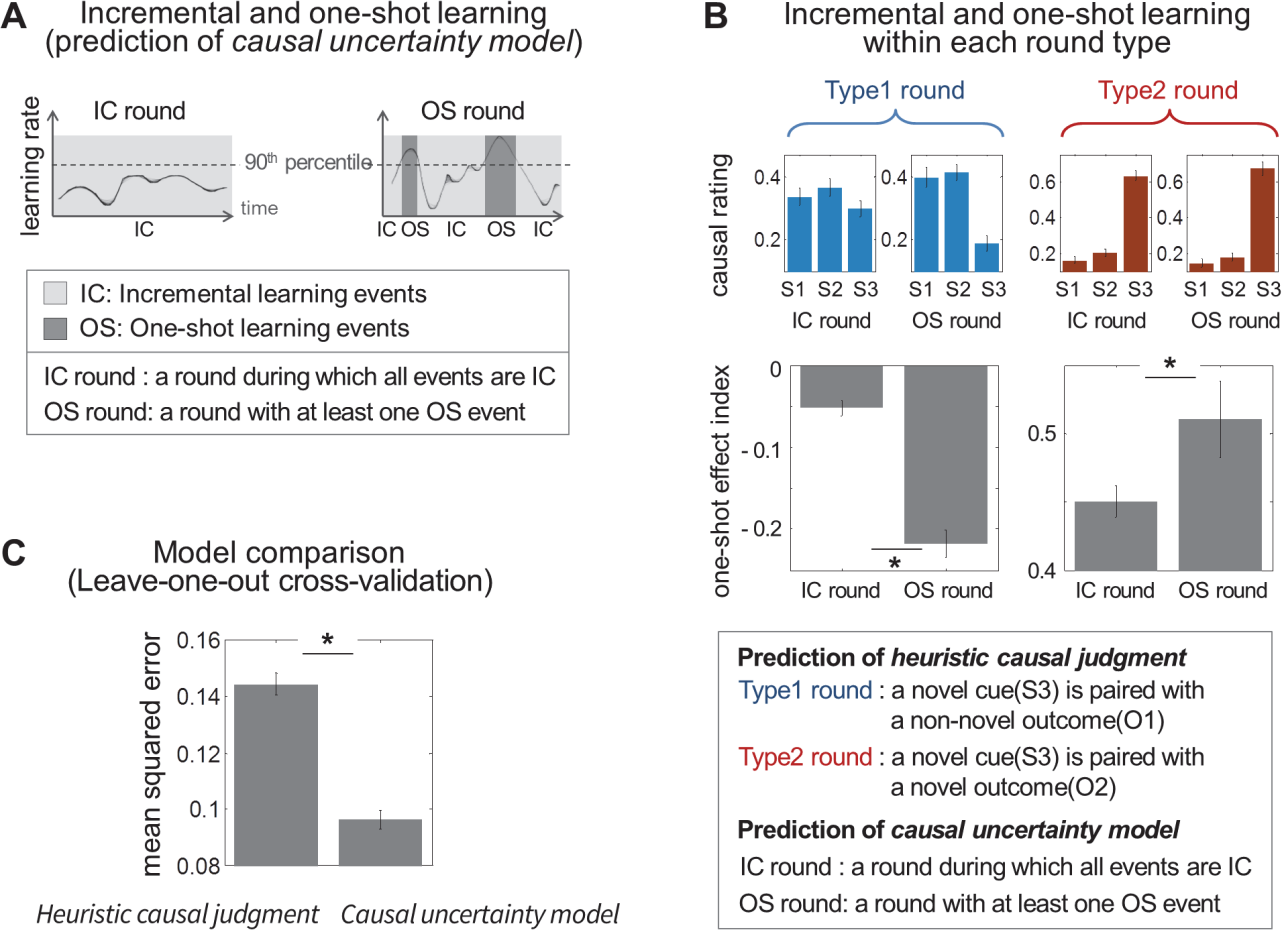
Model predictions. (A) Causal uncertainty model’s prediction about incremental and one-shot learning. Since the model provides event-by-event predictions about learning rate for each stimulus, we split events into two subtypes: “one-shot learning events” (OS), which refers to a stimulus presentation during which the model predicts rapid learning (>90th percentile) and “incremental learning events” (IC), which refers to a stimulus presentation during which the model predicts otherwise (<90th percentile). Accordingly, an “OS round” is defined as a round during which the model predicts occurrence of OS, and an “IC round” is defined as a round during which the model predicts no occurrence of OS. (B) The causal uncertainty model’s predictions that are beyond the predictions of the heuristic causal judgment, which draws a distinction between each round type—type1 and type2 rounds. For type1 rounds during which the model predicts occurrence of one-shot learning events (“OS round” in the type1 round), the corresponding one-shot effect index is significantly more negative than for type1 rounds during which the model predicts no occurrence of one-shot learning events (“IC round” in the type1 round), indicating that when one-shot learning occurs in the type1 rounds, with a high degree of certainty, participants attribute a novel outcome to non-novel stimuli, as opposed to the novel stimulus. On the other hand, for type2 rounds during which the model predicts the occurrence of one-shot learning events (refer to “OS round” in the type2 round), the corresponding one-shot effect index is greater than the type2 rounds during which the model predicts no occurrence of one-shot learning (refer to “IC round” in the type2 round). In both cases, one-shot effects are more dramatic when one-shot learning occurs during the round. *: *p* < 0.05; error bars are SEM across subjects. (C) Model comparison. Leave-one-out cross validation was used to validate the generalization performance of the models. The causal uncertainty model refers to the causal learning model proposed in the present study, and the heuristic causal judgment refers to the simpler heuristic model taking the stimulus-outcome novelty pairings into account assuming that causal uncertainty is high whenever stimulus and outcome novelty is high. *: *p* < 0.01; error bars are SEM across subjects.

Our causal uncertainty model and the heuristic causal judgment mechanism could both equally well account for the behavioral results reported above. To further distinguish the predictions of these models, we attempted to demonstrate that our causal uncertainty model makes additional predictions about the causal ratings above and beyond the distinction made by the novelty of the stimulus-outcome pair. To do this, we created event categories by stratifying stimulus events by the learning rates predicted by our causal uncertainty model: one-shot learning events (OS) are defined as a collection of discrete stimulus events during which the learning rate of the model is greater than the 90th percentile, while the remaining events are deemed to correspond to incremental learning events (IC) (see Fig 4A and Materials and Methods for more details); our independent model-based analysis indicated that the 90th percentile threshold is a viable predictor for distinguishing between one-shot learning and incremental learning (see S3 Fig for full details, including the rationale for the choice of the 90th percentile cut off). Second, we defined a one-shot learning round (OS round) as a round during which the model predicts occurrence of OS (see the right of Fig 4A) and the incremental learning round (IC round) as a round during which the model predicts no occurrence of OS (see the left of Fig 4A). It is important to note that the type1 rounds do not necessarily overlap with the IC rounds nor do the type2 rounds overlap with the OS rounds (see S4 Fig for more details).

In the type1 round during which a novel cue is paired with a non-novel outcome, the one-shot effect index (the causal rating for the novel cue minus the average causal ratings for the non-novel cues) for the OS rounds is more negative than for the IC rounds (see the left of Fig 4B; paired-sample *t* test, *p* < 0.01), demonstrating that the extent to which participants rated the non-novel stimulus as the most probable cause of the novel outcome in the OS rounds is greater than in the IC rounds. Conversely, in the type2 round, the one-shot effect index for the OS rounds is more positive than for the IC rounds (see the right of Fig 4B; paired-sample *t* test, *p* < 0.05), demonstrating that the extent to which participants rated the novel stimulus as the most probable cause of the novel outcome in the OS rounds is greater than in the IC rounds. These findings demonstrate that additional variability in the causal ratings can be explained by our causal uncertainty model to a greater extent than by the predictions of the heuristic causal judgment model (see S4 Fig for further predictions of the causal uncertainty model). This reiterates the point that one-shot learning is guided by causal uncertainty, which makes a distinction between the IC and the OS rounds rather than focusing on the novelty of the stimulus-outcome pair that distinguishes the type1 and the type2 rounds.

### Model Prediction Comparison

To further evaluate our hypothesis that a computation about causal uncertainty best explains the behavioral data, we formally pitted our proposed causal uncertainty model against the heuristic causal judgment model. We found that the model version formulating our hypothesis performed significantly better than the heuristic causal judgment model in terms of producing a smaller mean squared error of the difference between the model predictions and participants’ actual causal ratings using a leave-one-out cross validation procedure to take into account effects of model complexity and overfitting (paired-sample *t* test, *p* < 0.01; see Fig 4C and S5 Fig). The causal uncertainty model also exhibits rating patterns qualitatively more similar to subjects rating behavior than the alternative model (see Fig 4B for patterns of causal strength of the best model and S6 Fig for patterns of the heuristic causal judgment model). This result provides even stronger support for our contention that our causal uncertainty model provides a better account of participants’ one-shot causal learning behavior than a simple heuristic approach (also see S4 Fig for comparison between predictions of the causal uncertainty model and predictions of the heuristic causal judgment model).

### Additional Model Comparison

In addition, we also compared the performance of our causal uncertainty model against seven other alternative learning models typically used to account for incremental learning (See S1 Table for the full list of models). These include the Rescorla-Wagner model [6], the probabilistic contrast model [7], the Pearce-Hall associative model [8], variants of Bayesian latent variable models [47], a Bayesian causal network learning model allowing for an identification of a combination of stimuli as a cause of a particular outcome and for an establishment of a causal relationship between different outcomes [48], and a model of heuristic causal judgment that is designed to test the hypothesis that causal learning is driven by the novelty of the stimulus-outcome pair (see Materials and Methods). Among alternative models, the Bayesian causal network learning model proposes that participants might use a more complex causal structure, such as a combination of cues causally linked to outcomes, than that deployed in our causal uncertainty model. Our causal uncertainty model outperformed each and every alternative model, including the Rescorla-Wagner model showing the second-best model fitness, both quantitatively (paired sample *t* test at *p* < 0.05; see S5 Fig) as well as in terms of the qualitative ratings patterns (see S6 Fig). Collectively, these results provide further additional support for our model.

### Neural Computations Underlying Causal Learning

**Fig 5:**
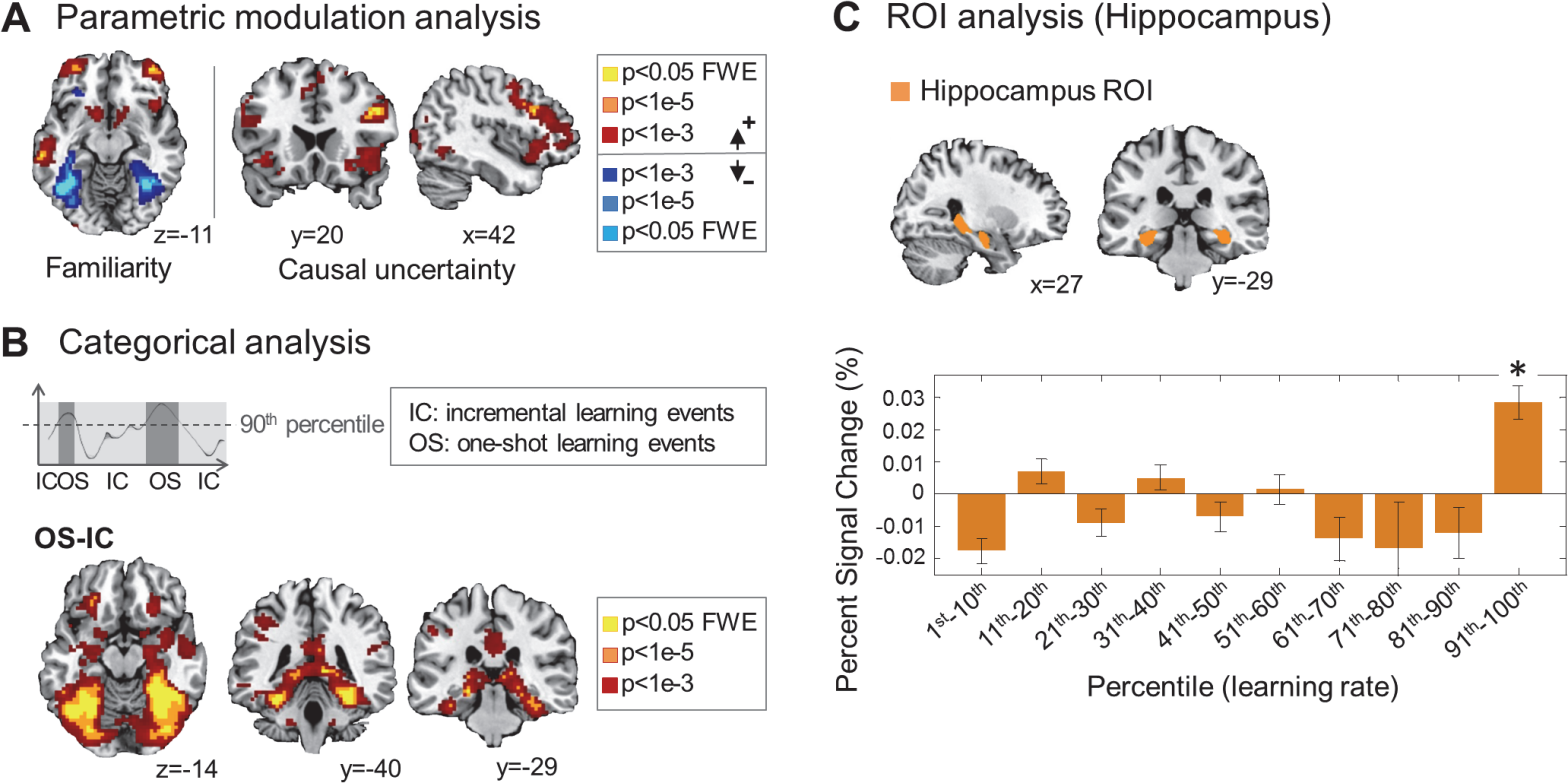
Neural correlates of one-shot-learning–related computations. (A) Neural substrates of familiarity and uncertainty processing in one-shot learning. Anterior lateral prefrontal cortex, dorsal prefrontal cortex, and parietal lobe encode signals associated with cue familiarity, whereas the fusiform gyrus encodes a cue novelty signal. Ventrolateral prefrontal cortex and parts of dorsomedial prefrontal cortex encode causal uncertainty signals. Effects significant at *p* < 0.05 (FWE corrected) are shown in yellow. (B) Involvement of the hippocampal memory system during one-shot learning. “One-shot learning events” is the collection of events during which the model predicts rapid learning (>90th percentile), and “incremental learning events” is the collection of events during which the model predicts otherwise (<90th percentile). Shown are significant categorical effects for one-shot learning events (OS) > incremental learning events (IC). We did not find any significant categorical effects for IC > OS, even with the liberal threshold *p* = 0.01 uncorrected. Effects are significant in fusiform gyrus, hippocampus, and parahippocampal gyrus. (C) Region-of-interest (ROI) analysis. We used an anatomically defined hippocampus to construct an ROI mask [54] (top), which includes CA1–CA3, dentate gyrus, and the hippocampal-amygdala transition area. Blood-oxygen-level dependent (BOLD) activity in the hippocampal ROI increases significantly in OS (91st–100th percentile of learning rate) but not in IC (1st–90th percentile) (paired-sample *t* test *p* < 1e-8). The asterisk shows the statistical level at which the signal change is significantly different from the baseline after Bonferroni adjustment for multiple comparisons across the ten learning bins (one-sample *t* test; *: *p* < 1e-6). Error bars are SEM across subjects.

To establish the neural computations underlying one-shot learning, we regressed each of our computational signals against the fMRI data (for testing signals, see Materials and Methods). For a strict identification of regions responsible for uncertainty processing, we first tested for regions correlating with stimulus novelty, which was defined simply as the number of times a participant had encountered a particular stimulus in the task (with a stimulus being most novel when first encountered). Next, we entered causal uncertainty into our analysis after adjusting for the effects of novelty so that areas found correlating with uncertainty are doing so above and beyond any effect of novelty. Novelty was positively correlated with activity in multiple areas—dorsal parts of prefrontal cortex, inferior parietal lobule, middle temporal gyrus (*p* < 0.05 family-wise error (FWE) corrected, Fig 5A; S2 Table), and Caudate (*p* < 0.05 cluster level corrected, S2 Table)—and was negatively correlated with activity in fusiform gyrus extending to the parahippocampal gyrus (*p* < 0.05 FWE corrected, Fig 5A; S2 Table). These results are highly consistent with previous findings implicating the medial temporal gyrus, parahippocampal gyrus, and fusiform gyrus in processing familiarity and novelty [49–53]. However, this interpretation might be complicated by the fact that, in our fMRI experimental design, the stimulus novelty could be associated with a growing association with a loss and a decreasing likelihood of being associated with the large novel gain.

Above and beyond novelty, causal uncertainty was found to correlate with activity in multiple prefrontal areas, including vlPFC (*p* < 0.05 FWE corrected, Fig 5A; S2 Table) and dorsomedial prefrontal cortex (*p* < 0.05 cluster level corrected, Fig 5A; S2 Table), consistent with our initial hypothesis.

### Selective Recruitment of Hippocampal System during Rapid Learning

To determine which brain regions are engaged on events during which the model predicts the participant will implement one-shot learning, we ran a categorical analysis between event types (Fig 5B). A significantly increased neural activation was found in multiple areas including hippocampus as well as fusiform gyrus (*p* < 0.05 FWE corrected, Fig 5B) on one-shot learning events compared to incremental learning events. We did not find any areas showing significantly increased activity on incremental learning events compared to one-shot learning events. One interpretation of these results is that neural systems previously implicated in incremental learning, such as the striatum [55,56], may always be active during all learning scenarios (including the one-shot case) but the hippocampal system is additionally recruited when one-shot learning needs to take place. The selective recruitment is not solely driven by the detection of a novel stimulus, which is a situation in which participants try to consciously remember a novel stimulus in order to establish strong stimulus-outcome associations, as indicated by the low correlation between the stimulus category of the best-fitting model (OS and IC) and the category by the novelty type (novel cue and non-novel cue) (Matthews correlation coefficient; mean = 0.29, standard deviation = 0.1 across subjects).

### Neural Computations Mediating Switching between Incremental and Rapid Learning

To further test our neural hypothesis about the role of hippocampus during OS, we subsequently ran an ROI analysis using an anatomically defined hippocampus mask [54]. The mean percent signal change, which quantifies how much the evoked BOLD response deviates from its voxel-wise baseline, was computed within the hippocampus ROI across all subjects. We found a significant increase in neural activity during OS but not during IC (paired-sample *t* test *p* < 1e-8, Fig 5C; also see S7 Fig for testing subregions of hippocampus). Importantly, when plotting hippocampal activity as a function of varying model-predicted learning rates throughout the experiment, we found evidence that the hippocampus is selectively recruited during very high learning rates (above the 90th percentile) and not for lower learning rates (*p* < 1e-6; one-sample *t* test after Bonferroni adjustment for multiple comparisons across the ten learning bins). This suggests that the hippocampus gets switched on at high learning rates, when one-shot learning needs to take place, and that the hippocampus is not engaged when more incremental types of learning take place. These results firmly support our hypothesis that the hippocampal memory system contributes to events during which one-shot learning takes place, further suggesting that this region is relatively silent during incremental learning.

**Fig 6:**
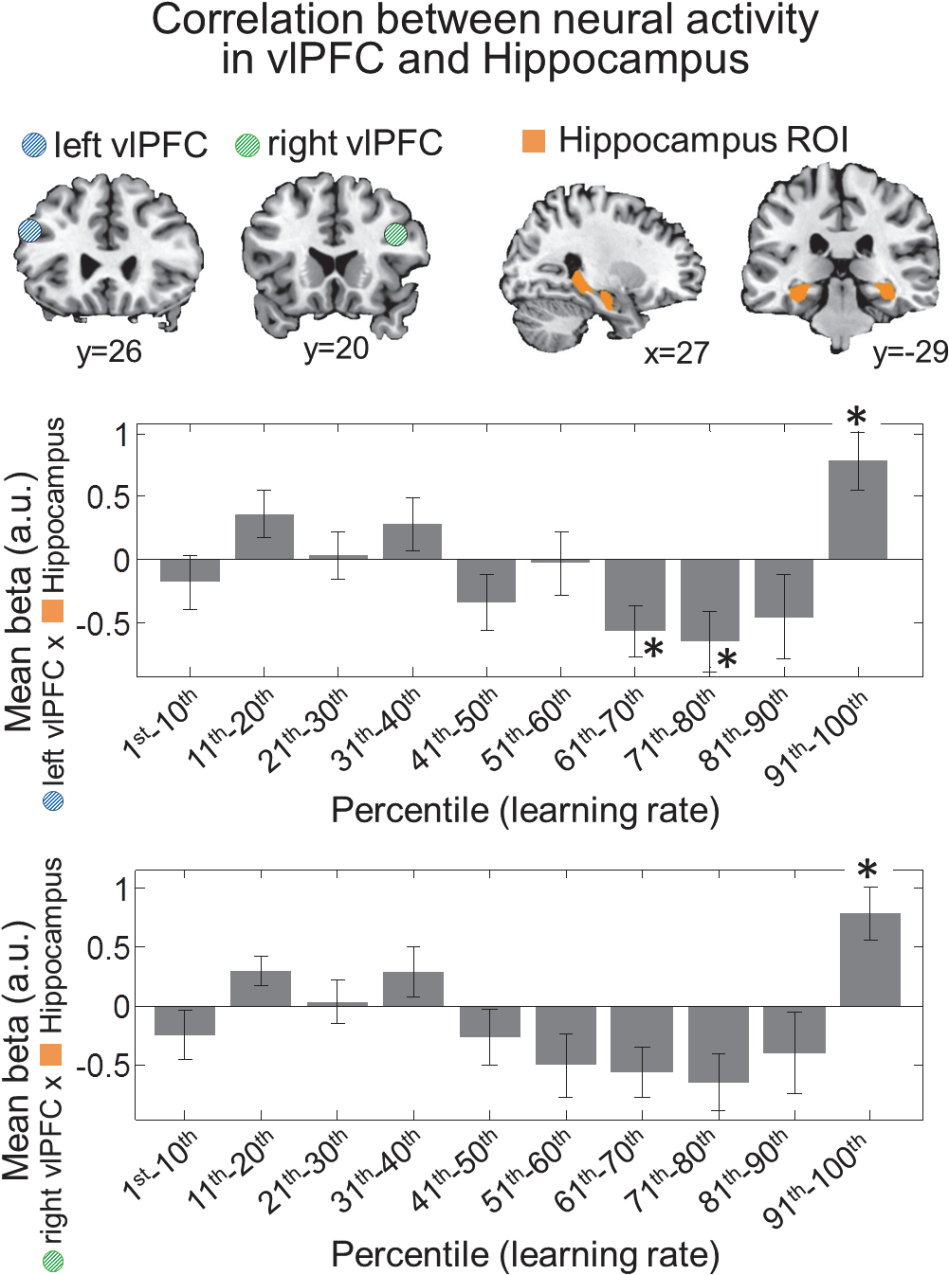
Functional correlation between prefrontal cortex and hippocampus activity. The blue and green circles represent a 5-mm sphere region of the left and the right ventrolateral prefrontal cortex, respectively, the area identified as processing causal uncertainty information, from which the first eigenvariate of BOLD signals were extracted. The left and right hippocampus ROIs were anatomically defined [54]. Shown are the average beta values within the hippocampus ROIs when the BOLD activity of ventrolateral prefrontal cortex was used as a parametric modulator in the fMRI analysis. This enables correlations to be calculated between activity in ventrolateral prefrontal and hippocampus as a function of learning rate. We found significant positive correlations between the two areas during the events in which rapid learning is predicted by the model (91st–100th percentile of learning rate). The asterisks shows the statistical level at which the beta value is significantly different from zero after Bonferroni adjustment for multiple comparisons across the ten learning bins (one-sample *t* test; *: *p* < 1e-2). Error bars are SEM.

In order to further characterize how the vlPFC, the region we found to most prominently encode causal uncertainty, interacts with the hippocampus during one-shot learning, we also ran a connectivity analysis (see Materials and Methods for technical details). We computed correlations between the neural signals in vlPFC and hippocampus for different learning rates and found that functional coupling between the vlPFC and the hippocampus was high during very high learning rates but not during learning rates associated with more incremental learning (Fig 6). Both the patterns of the vlPFC activation described above and of the connectivity results presented here indicate that the vlPFC may selectively interact with the hippocampus particularly under circumstances in which one-shot learning is warranted. This finding supports the possibility that the vlPFC may effectively operate as a switch to turn on the hippocampus when it is needed during one-shot learning situations and, furthermore, leads us to understand the nature of the activity in hippocampus during one-shot learning, i.e., that the hippocampus encodes causal uncertainty signal only during high learning rate events.

### Ruling out Alternative Explanations

We also tested whether our neural results might be accounted for by the following alternative factors.

#### Stimulus novelty

The first possibility is that hippocampus activity is solely driven by stimulus novelty, as opposed to causal uncertainty. This possibility is discounted for the following reasons.

First, the task was carefully designed to separate out the uncertainty from the novelty and also to separate out the effect of one-shot learning from novelty. The novelty constantly decreases with the repeated presentations of stimuli and outcomes, but the uncertainty does not. The average cross correlation between the novelty and the causal uncertainty regressor is 0.19 with a standard deviation of 0.09. This argument is again bolstered by the independent model comparison analysis (S5 Fig), demonstrating that our model, in which causal uncertainty contributes to the switching between the incremental and the one-shot learning, performed significantly better than other versions, including the Pearce-Hall model, in which stimulus novelty plays a key role in determining the learning rate. It is also noted that the change in the amount of uncertainty depends on how a stimulus is paired with different outcomes, whereas the novelty simply decreases with the number of occurrences of the stimulus presentation. Furthermore, the amount of uncertainty depends on multiple factors, such as the extent to which participants learn about the stimulus-outcome association from previous trials or the degree of confidence about the causality of the current stimulus compared to other stimuli (relative amount of uncertainty). Indeed, our task successfully combines these variables, creating variance enough to separate out the uncertainty from the novelty.

Second, for all of the neural analyses, effects of stimulus novelty were covaried out by including a novelty regressor in the general linear model in order to find areas correlating with causal uncertainty above and beyond the effect of novelty (refer to the previous section, “Neural Computations Underlying Causal Learning.” For full details, refer to Materials and Methods, “fMRI Data Analysis and GLM Design”), and causal uncertainty signal still survives the most stringent threshold (FWE *p* < 0.05; see both Fig 5A and S8 Fig). This strongly suggests that the activation patterns of hippocampus and its functional coupling with vlPFC reflect the influence of “causal uncertainty” of a stimulus on the neural process of switching between incremental and one-shot learning, as opposed to just the novelty of a stimulus.

#### Confound of learning rate with causal uncertainty

The second possibility is that the hippocampus activity, increasing with one-shot learning events compared to incremental learning events, and the vlPFC activity, correlated with the causal uncertainty regressor, are confounded because the learning rates are a beta transformation of causal uncertainty. This possibility is also excluded because the average cross correlation between the two regressors is 0.24, with a standard deviation of 0.04. One of the reasons that we have such a low correlation value is that the inverse temperature parameter value of our beta transformation of causal uncertainty is very high (refer to S1 Table); note that in principle, the correlation between a signal and its beta transformation roughly decreases with the value of the inverse temperature parameter and increases with the dimensionality of the beta transformation (= total number of stimuli).

#### Event novelty confound

The third possibility is that the classification of one-shot learning based on the model’s prediction about a learning rate might simply target the events that are the most novel. To test this, we separated out one-shot learning effects that occurred for non-novel stimuli and those that occurred for novel-stimuli. Approximately 40% of one-shot events occur for non-novel stimuli, thus showing that one-shot learning and novelty are not synonymous.

#### Stimulus novelty correlated with outcome value

The fourth possibility is that stimulus novelty might be negatively correlated with an outcome value in earlier trials. However, this possibility is discounted because our model predicts that the correlation between the two variables is low when one-shot learning occurs for non-novel stimuli; in other words, there would be a significant change in the outcome value predicted by the non-novel stimulus when the learning rate is high. This successfully dissociates the outcome value from novelty in the corresponding rounds. It is also noted that there are significant (either negative or positive) one-shot effects both in the type1 and the type2 round (refer to the one-shot effect index in Fig 3). We also checked if this correlation, albeit arguably less pronounced, has ever affected our results. We examined if there is an effect of the correlation (whether one-shot learning is predicted to occur for non-novel stimuli) on the causal ratings. We did not find any significant effects in this case (paired sample *t* test; *p* > 0.1), suggesting that whether or not the stimulus novelty covaries with an expected outcome value does not at all affect participants’ causal inference process. Furthermore, even if we assumed that there is a correlation, the effect would be nullified by the serial orthogonalization process of our general linear model [GLM] analysis meant to covary out the novelty effect (see Materials and Methods for full details). Collectively, there is little chance that the correlation prevails across rounds, and more importantly, this correlation does not at all influence our results.

#### Structure learning

Even though we showed in the earlier section that our model (causal uncertainty model) explains additional variability in the causal ratings above and beyond the predictions based on stimulus-outcome novelty expectations (heuristic causal judgment model), there is still a possibility that some participants might explore simple strategies for learning about a task structure, which would affect our results. To fully address this issue, we have taken the following steps:

We introduced two features to our task design to effectively prevent participants from pursuing a strategy of structure learning. First, before each experiment, we made sure participants understood that all rounds are independent of each other, that is, there is no general rule applied across rounds. This, combined with the incentivizing mechanism that is explained in the next section, leaves participants no choice but to make a fresh start each round. Additionally, after each experiment, we asked participants if they developed any strategies across rounds or noticed any dependencies between rounds while performing the task. None of our participants reported anything of the sort.

Second, participants were informed about the incentivizing mechanism (bonus round; refer to Materials and Methods, “Bonus round”) prior to the experiment so as to not only encourage them to do their best in every round but also to deter them from developing any structure learning strategy. The reason is that if participants simply assumed a dependent structure, and if this assumption were wrong, then the chance of winning would essentially decrease significantly (e.g., all of the stimuli chosen by the computer in the bonus round do not predict a positive outcome). Thus, there is little point in taking a risk of developing structure learning strategy and acting on it, against the information revealed by the task instructions. This incentive structure is thus expected to effectively reduce the expectation of dependency between rounds (i.e., a task structure or a heuristic method applied to the task).

We then ran post hoc analyses. We first checked if participants’ causal ratings exhibited one-shot learning effects in very early stages of learning, in which there is little chance of developing structure learning (S9A Fig). The one-shot effect index patterns for the early rounds are no different from the patterns for the rest of the rounds (paired-sample *t* test; *p* > 0.1), indicating that the one-shot learning effect exists even before structure learning would have been taking place. Next, we tested to see if participants gradually developed structure learning over the course of rounds, i.e., if there is a linear dependence between the one-shot learning effect and rounds (e.g., enhanced or reduced one-shot learning effect over time). We did not find any measurable effect of structure learning on causal ratings (S9B Fig, regression analysis; explanatory variable: round number; dependent variable: one-shot effect index).

We additionally carried out a model-based analysis to check if structure learning even occurred (S10 Fig). If there was a single cause (e.g., learned task structure) that affected participants’ behavior (causal ratings), then this should be reflected in the presence of a dominant causal association pattern across different rounds (statistical regularity). Thus, we quantified statistical regularity in the predictions about causal associations among different stimuli and between stimuli and outcomes, which underlie causal ratings in each individual round. We did not find in subjects’ causal rating patterns any evidence for the occurrence of statistical regularities (refer to S10 Fig for full details of our model-based analysis).

#### Novelty of stimulus-outcome pair

Our neural results might also be accounted for by alternative cognitive processes. One alternative hypothesis is that our neural results are driven by the novelty of the stimulus-outcome pair, i.e., the combination of a novel stimulus with a novel outcome as instantiated in the heuristic causal judgment model considered earlier. Once again, this possibility is ruled out by the fact that for all of the neural analyses, effects of the novelty of the stimulus-outcome pair were covaried out by including a categorical variable representing the pairing of novel stimuli and novel outcomes in the general linear model (For full details, refer to Materials and Methods, “fMRI Data Analysis and GLM Design”).

#### One-shot learning in hippocampus not driven by causal uncertainty

Another possibility is that one-shot learning effects in the hippocampus are caused by factors other than causal uncertainty. Though it may not be possible to demonstrate causality from our connectivity analysis, it is important to note that our inference about the relationship between vlPFC and the hippocampus is based on an extensive model comparison analysis, which dismisses many other alternative hypotheses, such as the Pearce-Hall model in which learning rates depend on stimulus-outcome associative strengths, the Heuristic causal judgment model incorporating stimulus-outcome novelty association, and a Bayesian inference model processing causal uncertainty information in the absence of learning rate control.

#### Activity differences between losses and gains

Yet another possible explanation is that the neural results are driven by the difference between losses (which are the novel outcome) and gains (which are the non-novel outcome) in our fMRI experiment. To address this possibility, in a follow up fMRI analysis we compared activations to receipts of losses versus gains. We found no significant activation in the hippocampus or vlPFC in this analysis even at *p* < 0.001 uncorrected, suggesting that our fMRI results are not simply driven by the difference in the sign of the novel versus non-novel outcomes. However, since our fMRI task only tests for effects of outcome novelty in the loss domain, further work will be needed to fully address this issue.

#### Additional sequential presentation effects

We also investigated effects arising from a sequential presentation of multiple stimuli on causal beliefs by introducing a few parameters to each of the possible model types. First, we introduced two parameters to take into account that, when participants recall a list of presented stimuli to update causal beliefs after an outcome presentation, they tend to recall the first or the last stimuli best (for full details, refer to Materials and Methods, “Additional parameters—Primary and recency effect”). The fitted parameter values of the model are significantly greater than zero, indicating that both the first and the last presentations of stimuli contribute to the update of the causality more than others do. (The mean and the standard deviation of the primacy weight are 0.36 and 0.75, respectively; one-tailed one-sample *t* test; *p* = 0.0005. The mean and the standard deviation of the recency weight are 0.37 and 0.83, respectively; one-tailed one-sample *t* test; *p* = 0.001). This is consistent with previous findings about the effect of a serial position on recall performance [57,58].

Second, we tested two different versions of each model type to see if participants take the number of presentations of each stimulus into account (additive effect) or if they only take the presence or absence of the stimulus into account when they update the causal beliefs based on causal uncertainty (sole effect; for full details, refer to Materials and Methods; S1 Table illustrates how these effects were tested for each individual model). The version implementing the additive effect overall outperforms the version implementing the sole effect (paired-sample *t* test; *p* < 0.05), though the sole effect fit better for a few participants (see S5A Fig for the summary of the comparison between the two effects).

## Discussion

Here we provide a novel computational account for one-shot causal learning, which is a phenomenon that a variety of incremental learning models fail to explain. Our computational analysis of behavior reveals that humans take into account the relative uncertainty about the causal relationship between stimuli and different outcomes in order to drive rapid learning. Our findings about the role of causal uncertainty in learning is partly in agreement with previous theoretical proposals that learning rate is modulated based on detecting changes in environmental volatility or jumps in contingencies [8,13,14]. By contrast, the present model institutes very rapid increases in learning rate for causal relationships that are highly uncertain, in a manner that can reproduce one-shot learning. Moreover, while previous models of probabilistic causal inference addressed issues concerning how causal knowledge, including uncertainty, is learned and represented [10–12], such approaches have not focused on how causal uncertainty engages with causal strength in order to implement one-shot learning. Thus, our current model is the only framework to date that is optimized to account for one-shot learning.

### Critical Contribution of the Prefrontal System to a Causal Learning Process

At the neural level, our findings indicate evidence for involvement of a very specific neural system for the range of learning rates that would support one-shot learning according to our model. Specifically, activity in the hippocampus was ramped up for high learning rates (90th percentile or more) relative to slower learning rates, in which, by contrast, the hippocampus showed no activity. Thus, the hippocampus appeared to be recruited in a switch-like manner, coming on only when one-shot learning occurred and being silent otherwise.

Our findings support the theoretical proposition that episodic memory systems play a unique role in guiding behavior, distinct from the contribution of other systems involved in more incremental types of learning such as goal-directed and habitual instrumental control and Pavlovian learning [15,59]. It is worth noting that while previous studies have found hippocampal involvement in goal-directed learning, such as in our recent study in which the hippocampus was found to respond during the “planning” stage of a goal-directed action [60], it is possible that the hippocampus is involved in contributing to two distinct computational processes. It is still an open question as to whether the mechanisms involved in one-shot learning are conceptually and neurally distinct from those being studied in goal-directed learning.

The fMRI results suggest that parts of the prefrontal cortex including the vlPFC are involved in encoding uncertainty about the causal relationships between cues and outcomes. The ventrolateral prefrontal cortex has previously been implicated in memory encoding and explicit memory attribution [40,50,61–64]. Our findings provide new insight into the nature of the top-down control functions of vlPFC. In our computational model, the degree of causal uncertainty surrounding a cue-outcome relationship is used to dramatically adjust the learning so as to engage one-shot learning when required. The vlPFC was found to encode the causal uncertainty signal that in turn could be used to modulate learning rates, in a highly nonlinear fashion.

### Selective Interaction between Prefrontal and Hippocampal System during One-Shot Learning

One interpretation of these findings is that vlPFC uses knowledge about causal uncertainty to act as the controller of a switch, engaging episodic memory systems when learning needs to proceed from a single episode (one shot) as opposed to incrementally. This view is supported by our demonstration of the interactions between vlPFC and the hippocampus. A previous study found enhanced connectivity between the vlPFC/ dorsolateral prefrontal cortex (dlPFC) and the hippocampus in correct memory retrieval [64], and this finding invited the speculation that lateral prefrontal cortex is recruited to guide explicit associative memory decisions. The present study greatly extends this proposal by providing a specific computational account for how connectivity between the two regions is controlled: the present results suggest that change in learning rate is an important factor involved in governing the degree of connectivity between these areas during one-shot learning. When learning rates were high (above the 90th percentile), there was increased connectivity between vlPFC and hippocampus, which also corresponded to the selective increase in activity in the hippocampus during task performance, consistent with the possibility that vlPFC is acting to engage the hippocampus when it is required to facilitate one-shot learning.

It is important to note that not all types of one-shot learning may be mediated by the hippocampus nor is the hippocampus likely the sole contributor to this process. In particular, taste aversion learning may depend on additional neural circuits [65,66], and there is ongoing debate about whether or not the hippocampus is even necessary for taste-aversion learning [67]. Here, the outcomes used in the present task (small monetary gains and losses) are relatively inconsequential, as compared to taste aversion learning or learning with other highly biologically relevant outcomes. One important extension of the present work would be to also examine one-shot learning in circumstances involving more biologically relevant stimuli such as aversive tastes or pain in order to ascertain whether similar or distinct neural structures are implicated. In future work, it would also be worthwhile to determine the extent to which anxiety-related mechanisms might modulate hippocampal activity during one-shot learning when individuals are presented with highly aversive stimuli.

### Integrated Neural Computations for Rapid Causal Learning

In the present work, we also attempted to provide a specific computational account and to discount alternative explanations for our data. One very obvious possible alternative account is that participants may simply use a heuristic strategy in which the most novel stimuli are assumed to be responsible for causing any given outcome, as opposed to using the more sophisticated strategy of representing the causal uncertainty about a stimulus-outcome relationship. However, when we directly tested this heuristic strategy against out behavioral data, it did not account as well for the behavioral results as did our causal uncertainty model. Furthermore, we tested a number of other alternative models that are traditionally used to account for incremental associative learning. In all cases, the model we proposed was superior. This suggests that the present model is a highly parsimonious one with two core elementary features that are likely to be important elements of how the brain solves the problem of one-shot learning: the first is a computation of a representation of uncertainty about the causal relationship between events, and the second is the flexible adjustment of learning rates to accommodate rapid learning about those events. Those two key model features that correspond to the two mains signals we observed in the brain during task performance are, we suspect, likely to be an important component of any successful algorithmic approach to one-shot learning.

The predictions of the present model together with the neural findings warrant an investigation of more challenging problems, including whether or not one-shot learning would occur when the amount of causal uncertainty keeps increasing in spite of a continual decrease in stimulus novelty. This refers to highly chaotic situations in which making observations does not necessarily guarantee resolving uncertainty in the causal relationships between stimuli and outcomes. In future work, testing for such effects would allow us to investigate how the causal inference process breaks down in such conditions.

Taken together, these findings form the basis of a new understanding of the neural computations underlying the ability to learn from a single exposure to an event and its consequences. Developing a detailed account of when rapid learning takes place and which brain areas are engaged in this process might subsequently open the window to better understanding situations under which rapid causal attributions are generated in a dysfunctional manner such as in misattribution, superstition, and delusional reasoning [2–5,68].

## Materials and Methods

### Participants

Forty-nine adult participants (14 females, between the ages of 19 and 43, mean = 25.8, standard deviation = 5.2) were recruited in total. Two participants, who gave the same unchanging causal ratings in most of the trials, were excluded from our analysis. Out of these, 20 (ten females, between the ages of 19 and 40, mean = 26.1, standard deviation = 5.3) participated in the fMRI study (experiment 1), 13 participants (two females, between the ages of 20 and 43, mean = 25.5, standard deviation = 6.2) participated in experiment 2 (behavioral only), and 14 participants (two females, between the ages of 22 and 35, mean = 25.8, standard deviation = 4.4) participated in experiment 3 (behavioral only).

### Ethics Statement

All participants gave written consent, and the study was approved by the Institutional Review Board of the California Institute of Technology (Protocol number 12–359). Participants were screened prior to the experiment to exclude those with a history of neurological or psychiatric illness.

### Experiment 1 (fMRI)

#### Task instruction

During the 100-min fMRI experiment, 20 participants were asked to complete 40 rounds. Each round comprises a learning phase and a rating phase. The separation of the learning and the rating phase is intended for minimizing interference from motor actions or any unwanted interaction with the rating procedure, thereby allowing us to purely measure neural responses related to learning during the learning phase. Before the experiment began, participants were told that in the task on each trial they will be presented with a sequence of pictures that will vary in the degree of frequency in which they are presented, followed by receiving a monetary outcome of either winning or losing a certain amount of money. They were also told that after several rounds of trials they will be asked to make several ratings about the stimuli they saw and that rounds are independent of each other. They were also told that after they complete all rounds, they can take away cumulative winnings, which will depend on the amount of money they win on each trial.

#### Learning phase

The learning phase consists of five trials of stimulus presentations followed by an outcome presentation. There is no pretraining session outside the scanner. There are five presentations of stimulus pictures within each trial. In each trial, participants are presented with a sequence of pictures (fractal images) one at a time, followed by presentation of an outcome. Each stimulus is displayed for 1 s. The presentations of successive stimuli are separated by a variable temporal interval drawn from a uniform distribution between 1 to 4 s. The outcome is displayed for 2 s. The stimulus pictures vary in the degree of frequency in which they are presented; among 25 presentations in the five trials, the most frequently presented pictures are presented 16 times (non-novel stimuli), the second-most-presented ones are shown eight times (non-novel stimuli), and the novel pictures are presented one time (novel stimuli). After the sequence of pictures has been presented, participants receive a monetary outcome. This outcome is in some cases to win a certain amount of money (10 USD) or else on other occasions to lose a certain amount of money (-50 USD). The winning amount of money is presented in four trials (non-novel outcome), and the losing amount of money is presented in one trial (novel outcome). It takes 20 s on average to complete a single trial; this means that the longest delay between a given stimulus and a subsequent outcome is 20 s. The intertrial intervals are randomly sampled from a continuous uniform distribution with a minimum of 1 s and a maximum of 4 s.

#### Rating phase

After the fifth trial, the rating phase begins. The rating phase consists of three subphases. The first subphase begins with a liking rating: “How much do you like this picture?” (hedonic rating). Each stimulus is presented in turn, and participants make a rating based on their subjective feelings about how much they like the picture. The rating bar is shown on the screen below the picture using a scale from -5 (“dislike”) to 5 (“like”), with a step size of 1. The order of stimulus presentations was randomized. In the second subphase, participants are asked to make ratings about how likely it is that each individual stimulus encountered during the round can cause a novel outcome (“causal rating”): “How much do you think this causes a good/bad outcome?” Specifically, each stimulus-outcome pair is presented in turn, and participants rate the causal relationship between the stimulus and the outcome on a scale from 0 (“not at all”) to 10 (“very likely”), with a step size of 1. The order of those ratings and the order of stimulus presentation was randomized. A maximum of 4 s is allowed for each submission. In the third subphase, three of the stimuli that participants saw over the course of that particular round are presented as vertices of a triangle, and participants are asked to move a little cursor (“o” mark) inside the triangle by using the left or the right key. The position of the cursor inside the triangle determines the likelihood or probability that the computer will select that picture for inclusion in a bonus round at the end of the experiment, in which the computer computes the amount of money won or lost. Participants need to move the cursor (either left, right, up, or down) to the location corresponding to their beliefs about how likely it is that this particular stimulus caused the bad outcome. Subjects are motivated to move the cursor away from the location of the stimuli they think caused the bad outcome. Conversely, the closer they move the selection point toward one of the stimuli, the higher the chance they will get that stimulus in the bonus round, and the higher the chance the computer will discard other stimuli. They then press “y” to submit the selection. A maximum of 8 s is allowed for each submission. Each time subjects are late or fail to submit a selection, 1 USD is deducted from the total amount subjects earn at the end of the experiment. Prior to the experiment, participants were instructed to summarize the causal ratings submitted in the second subphase prior to the experiment in the third subphase to make them aware that they need to be consistent when submitting causal ratings. This was intended to ensure that an incentive-compatible scheme applied to both types of causal ratings.

#### Bonus round

At the end of the experiment, the bonus round is run. In this round, the computer randomly picks three rounds from the main session and then selects three stimuli from those rounds (one stimulus per round). The probability with which a particular stimulus is selected by the computer for inclusion in the bonus round depends on the rating participants made in the third rating subphase, as those stimuli given a higher causal rating in the selected round are chosen more frequently by the computer (proportional to the probabilities assigned by the participants as determined by the location of the cursor within the triangle on that trial). During the bonus round, once the selected stimuli are presented, participants can win or lose money on that bonus round depending on the pictures that were presented and the outcomes that are delivered. This bonus round is intended to implement an incentive-compatible belief elicitation, thereby ensuring that participants are motivated to give accurate ratings. Participants received the payouts they earned after the bonus round. In order to maximize the chance of winning, participants need to be accurate about the rating and consistent about the ratings in each rating subphase. The ratings that participants submitted in the second subphase and in the third subphase turned out to be highly correlated for all participants (median correlation coefficient = 0.66; correlation coefficient test; *p* < 0.01 for all 47 participants), suggesting that even in the second subphase, in which a simple causal rating was elicited (as opposed to a probabilistic belief), the participants were motivated to provide accurate ratings. However, because of the complexity of the submission process of the third phase, some participants reported difficulty in submitting accurate ratings in that subphase. This led us to conclude that the ratings from the second subphase are the most informative, and hence, this causal rating data was used for all subsequent analysis.

#### Stimuli

The image set for the stimuli consisted of 127 fractal images. In order to indicate an outcome, the outcome was shown by illustrating a numerical amount depicting the amount won or lost. When beginning each round, the stimulus computer randomly chose three fractal images from the set of fractals, excluding the fractals already shown in previous rounds. The chosen images were subsequently used to represent each stimulus. We imposed a constraint on image presentation, such that the novel images (novel stimulus and novel outcome) are presented in the last two trials. The reason for doing this is so that participants build up enough experience of the non-novel images in the earlier trials within a round. Specifically, when generating 25 stimuli presentations, we first generate the first, the second, and the third fractal image 16, eight, and one time(s), respectively. We then perform a random permutation of the integers from 1 to 25 inclusive for the first and the second images and 16 to 25 inclusive for the third image. The same principle is applied to outcomes, with the type of outcome (novel versus non-novel) following a pseudorandom order.

We created two round categories by manipulating the novelty of the stimulus-outcome. Each round type considers a case in which a novel cue is paired with a non-novel outcome (type1 rounds) or a case in which a novel cue is paired with a novel outcome (type2 rounds). It is important to have the two round types contain an equal number of rounds because each participant performs only 40 rounds in total. To preclude unbalanced round types, our stimulus program implemented a pseudorandomization process that iteratively generates stimuli and outcomes until satisfying the condition that round types are equally balanced.

### Experiment2 (Behavioral)

The design was the same as that of experiment 1 except that we used two kinds of outcome pairs (non-novel, novel): (10,-50) and (50,-10). Thirteen participants were asked to complete 40 rounds. The change of the outcome pair was made on a trial-by-trial basis. This allows us to test the effect of outcome amount on subject ratings and to see if the one-shot learning effect still stands up.

### Experiment3 (Behavioral)

The design was the same as that of the experiment 1 except that we used two kinds of outcome pairs (non-novel, novel): (10,-50) and (-10,50). Fourteen participants were asked to complete 40 rounds. The change of the outcome pair was made on a trial-by-trial basis. This allows us to test the effect of the sign of an outcome on subject ratings and to see if the one-shot learning effect still stands up.

### Image Acquisition and Processing

Functional imaging was performed on a 3T Siemens (Erlangen, Germany) Tim Trio scanner located at the Caltech Brain Imaging Center (Pasadena, California) with a 32-channel radio frequency coil for all the MR scanning sessions. To reduce the possibility of head movement related–artifacts, participants' heads were securely positioned with foam position pillows. High-resolution structural images were collected using a standard MPRAGE pulse sequence, providing full brain coverage at a resolution of 1 mm × 1 mm × 1 mm. Functional images were collected at an angle of 30° from the anterior commissure-posterior commissure (AC-PC) axis, which reduced signal dropout in the orbitofrontal cortex [69]. Forty-five slices were acquired at a resolution of 3 mm × 3 mm × 3 mm, providing whole-brain coverage. A one-shot echo-planar imaging (EPI) pulse sequence was used (TR = 2800 ms, TE = 30 ms, FOV = 100 mm, flip angle = 80°).

### fMRI Data Analysis and GLM Design

The SPM8 software package was used to analyze the fMRI data (Wellcome Department of Imaging Neuroscience, Institute of Neurology, London, United Kingdom). The first four volumes of images were discarded to avoid T1 equilibrium effects. Slice-timing correction was applied to the functional images to adjust for the fact that different slices within each image were acquired at slightly different points in time. Images were corrected for participant motion, spatially transformed to match a standard echo-planar imaging template brain, and smoothed using a 3-D Gaussian kernel (6-mm FWHM) to account for anatomical differences between participants. This set of data was then analyzed statistically. A high-pass filter with a cutoff at 129 s was used.

A GLM was used to generate voxelwise statistical parametric maps (SPMs) from the fMRI data. We created subject-specific design matrices containing the regressors (R) in the following order: (R1) a block regressor encoding the average BOLD response during the full duration of the rating submission phase, (R2) a regressor encoding the average BOLD response at the time of each stimulus presentation (1-s duration), (R3) a parametric modulator encoding the novelty of stimuli, (R4) a parametric modulator encoding the posterior variance (refer to section “Bayesian inference for causal learning: (2) Latent inhibition”), (R5) a regressor encoding the average BOLD response at the outcome state (2-s duration), and (R6) a categorical variable representing the novelty of the stimulus-outcome pair at the time of each outcome presentation (1 if both the stimulus and the outcome is novel, 0 otherwise). The order of the regressors is determined in a way that eliminates the variance of no interest. These regressors were orthogonalized with respect to the previous ones in order to prevent shared variance from being explained multiple times.

### Whole-Brain Analyses

All of the findings we report survive whole-brain correction for multiple comparisons at the cluster level (corresponding to “+”; height threshold *t* = 3.53, extent > 100 voxels, *p* < 0.05 corrected). We used this single basic statistical threshold throughout the analysis. The areas surviving the most stringent threshold, *p* < 0.05 FWE whole-brain corrected at the voxel level, are marked with “*” in S2 Table and also shown in Fig 5 (cyan and yellow blobs in the statistical maps). In addition, for all the figures, in order to show the full extent of the activations, we used the following stratification: *p* < 0.05 FWE, *p* < 1e-5 uncorrected, and *p* < 1e-3 uncorrected.

### Categorical Analysis

We define two types of events: OS are defined as a collection of stimulus events in which the learning rate of the model is greater than the 90th percentile, while the rest of the events are deemed to correspond to IC. The trials in which a novel cue is presented with a novel outcome amount to 10% of total trials (1/2 type2 round x 1/5 of novelty-matching trials = 1/10), and during these trials the learning rate almost always rises to peak. Thus, we define one-shot learning threshold as the learning rate of 90th percentile.

### Physiological Correlation Analysis

To test whether there is a functional coupling between the prefrontal area associated with uncertainty processing and the hippocampus modulated by the learning rate, we performed a physiological correlation analysis. The procedure is the same as a psychophysiological interaction analysis [70] except that the psychological variable is a combination of multiple boxcar functions, whose interval is given by the percentile of learning rate. We used the first eigenvariate of BOLD signals from the ventrolateral prefrontal cortex extracted from a 5-mm sphere centered at the coordinates of the cluster identified as correlating with causal uncertainty (S2 Table). The extracted BOLD signal was deconvolved in order to retrieve the underlying neuronal signal. The deconvolved signal was then used as a parametric regressor for the GLM analysis. The onset times for this first parametric regressor correspond to a collection of events during which the learning rate is between the 1st and 10th percentile. After performing the GLM analysis, the average beta value is computed within the anatomically defined bilateral hippocampus ROI [54]. This analysis is repeated for each size bin (10th percentile). The average beta value represents correlation between the neural activity of the ventrolateral prefrontal cortex and hippocampus during the events whose intervals are given by binned percentile of learning rate.

### Effect of Orthogonalization

To see if our fMRI findings are an artifact of the order in which the regressors had been entered into the fMRI design matrix because of serial orthogonalization, we ran another GLM analysis in which the main regressors are not orthogonalized with respect to each other (i.e., by disabling serial orthogonalization). The results are summarized in S8 Fig. All of the results are highly consistent with what we have reported in our main results and survive corrected thresholds. This indicates that our main results are very robust to orthogonalization order.

### Model Comparisons

The followings are the list of the models used for model comparison (refer to S1 Table for full details):

#### Heuristic causal judgment

The heuristic causal judgment model permits a test of the hypothesis that a novel stimulus is rated as the most probable cause of a novel outcome if that association is consciously remembered and detected. It accommodates the fact that the causal ratings for the novel stimulus are greater than for the non-novel stimulus in the type2 rounds. Specifically, the ratings for the non-novel stimulus are sampled from a discrete uniform distribution between 0 and 5 and for the novel stimulus between 6 and 10 if the novel stimulus is paired with a novel outcome. These parameters are determined to match the sample means of the distributions and the average ratings of participants in each condition. The ratings are drawn from a discrete uniform distribution between 0 and 10 in type1 rounds.

#### Rescorla-Wagner model (Delta rule model)

The Rescorla-Wagner (Delta-Rule) model was inspired by the Rescorla-Wagner model (R-W) of classical conditioning [6]. The model updates the associative strength between a conditioned stimulus and an unconditioned stimulus based on the discrepancy between a predicted and actual event. Specifically, the delta rule [6,71] is used to make a prediction about an outcome given the stimulus.

$$V^{\left(t+1\right)}=V^{\left(t\right)}+\alpha \gamma (\lambda -V_{total}^{\left(t\right)}),$$ (1)

where *V* ^(*t*)^ is the associative strength between a stimulus and an outcome at trial *t*, and α,γ is the salience of the stimulus and the learning rate, respectively. λ is the magnitude of outcome, and $V_{total}^{\left(t\right)}$ is the sum of associative strengths for all stimuli available at trial *t*. The model assumes that the learning rate is a constant and repetitive presentation of a stimulus increases its salience. Although this class of model has been shown to successfully account for numerous phenomena related to classical conditioning, some failures have been reported because of the way it deals with novel stimuli. For example, the model fails to learn the association correctly if a novel stimulus is a conditioned inhibitor or has an innate bias for prediction [72]. Moreover, the assumption that the learning rate remains constant renders the model incapable of implementing a transition between slow and rapid learning.

#### Probabilistic contrast model

The probabilistic contrast model (PCM) [7] provides a probabilistic evaluation of the causal relationship between a cause (C) and an effect (E):

ΔP=P(E|C)−P(E|∼C), (2)

where *P*(*E*|*C*) is the probability of *E* given *C*, and *P*(*E*|∼*C*) is the probability of *E* in the absence of *C*. The model allows that a linear combination of causes acts on an outcome effect. This model is equivalent to the power PC theory under the assumption that the probability of the outcome effect in the absence of the cause is zero [73].

#### Pearce-Hall model

The Pearce-Hall model [8] implements control of a learning rate according to the degree of discrepancy between the magnitude of an actual outcome and the sum of associative strengths in a preceding trial. The less an outcome is predictable given the stimulus, the more rapidly the model learns about the stimulus and the expected outcome. The learning rate is assumed to be high for a novel stimulus. The model updates the associative strength between a stimulus and an outcome as follows:

$$V^{\left(t+1\right)}=V^{\left(t\right)}+\alpha \gamma \lambda ,$$ (3)

where *V* ^(*t*)^ is the associative strength between a stimulus and an outcome at trial *t*, α represents the intensity(salience) of a stimulus, and λ is the magnitude of an outcome. The learning rate *γ* is computed as follows:

$$\gamma =\left|\lambda -V_{total}^{\left(t\right)}\right|,$$ (4)

where λ is the magnitude of an outcome, and $V_{total}^{\left(t\right)}$ is the sum of associative strengths for all stimuli available at the trial *t*. The characteristics of this model are similar to McLaren’s association model [9]. Specifically, a high learning rate is assigned to the model in early stages of learning, whereas in later stages, the learning rate declines to prevent overshoot. The model assumes that learning is driven exclusively by stimulus novelty, the number of times a particular stimulus has been presented. There is no need to test McLaren’s model because in our task the predictions of the model are the same as the Pearce-Hall model.

#### Bayesian causal structure learning

In order to evaluate the hypothesis that participants might learn to establish a complex causal chain by taking all possible associations among different stimuli and between stimuli and outcomes into account, we test the online Monte Carlo Markov Chain–based learning model [48,74]. The model allows for identification of a combination of stimuli as a cause of a particular outcome and for a further establishment of a causal relationship between different outcomes. This model estimates the posterior over causal Bayesian network structures, in which each node represents a cue or an outcome and the corresponding causal structure is defined as a directed acyclic graph. The causal strengths between each stimulus and each outcome node correspond to causal ratings. The number of steps to take before drawing samples (burn-in) and the number of samples to draw from the chain after the burn-in are optimized to account for participants’ rating patterns [48].

#### Bayesian inference for causal learning: (1) Latent class model

Although a hierarchical Bayesian model advances the theory of causal inference to give more detailed description of how prior knowledge guides causal inference [10], it does not provide a full specification of when and how one-shot learning occurs. Here we test an alternative hypothesis that the modulation of learning rate occurs based on uncertainty in the causal relationship between a stimulus and an outcome (causal uncertainty), as opposed to the pure novelty of a stimulus. The model is based on Bayesian machinery, which endows us with a formal way to estimate the amount of causal uncertainty (posterior variance) and the causal strength (posterior mean). However, the model departs from Bayesian inference because it is assumed that the learning rate is a function of causal uncertainty, as opposed to a constant.

We use a finite mixture model [47], the simplest form of latent class model, because only two types of outcomes are available and this information is fully informed prior to the experiment. We assume that events are conditionally independent [47]. The conditional probability of each stimulus causing a particular outcome is given by the following:

$$P(C|\theta )=\{\begin{array}{c} \theta_{1}\\ \theta_{2}\\ \theta_{3} \end{array}\;\begin{array}{c} if\;C=S_{1}\\ if\;C=S_{2}\\ if\;C=S_{3} \end{array},$$ (5)

where *C* represents the cause of an outcome, *S*_*i*_ represents the i-th stimulus, *C* = *S*_*i*_ refers to the case in which the outcome is caused by *S*_*i*_, and *θ* is a prior probability with $\sum_{i=1}^{3}\theta_{i}=1$.

It is further assumed that *θ* is drawn from a prior distribution:

$$(\theta_{1},\theta_{2},\theta_{3})\;∼\;\text{Dir}(\lambda_{1},\lambda_{2},\lambda_{3}),$$ (6)

where Dirichlet refers to a Dirichlet distribution with $\sum_{i=1}^{3}\lambda_{i}=1$. The initial prior was assumed to be uniform.

Suppose that T discrete events *D* = {*S*_1_,*S*_3_,…,*S*_1_} were observed. It follows that the posterior *θ*|*D* (the prior conditioned on the evidence *D*) is also Dirichlet (conjugacy):

$$\text{Dir}(\alpha_{1},\alpha_{2},\alpha_{3}),$$ (7)

where α_*i*_ = λ_*i*_ + *x*_*i*_ with *x*_*i*_ being the salience of the stimulus *S*_*i*_. The simplest definition of *x*_*i*_ is the number of occurrence of the presentation of the stimulus *S*_*i*_ given all events are equally effective. Then the update rule for the posterior *p*(*θ*|*D*) is Δα_*i*_ = γ_*i*_ *x*_*i*_, with γ_*i*_ being the learning rate for the stimulus *S*_*i*_; Δα_*i*_ = −γ_*i*_ *x*_*i*_ when the stimulus is paired with the other type of outcome. The learning rate determines the extent to which the effect of presentation salience is amplified. The learning rate values are updated for each stimulus after each trial, so they do not change within a trial.

Note that in order to prevent the Bayes model from breaking down, we added a boundary to the algorithms so as to not allow negative alpha values (alpha = max(0,alpha)); if it reaches zero or negative, then it stays at zero until it gets a positive amount of update. This means that the model will not learn any more when there is no remaining amount of uncertainty left in the causal relationship.

#### Bayesian inference for causal learning: (2) Latent inhibition

The mean and the variance of the posterior are given as follows:

$$E(\theta_{i}|D)=\frac{\alpha_{i}}{\alpha_{0}}\;\text{and}\;Var(\theta_{i}|D)=\frac{\alpha_{i}(\alpha_{0}-\alpha_{i})}{\alpha_{0}^{2}(\alpha_{0}+1)},\;i=1,2,3,$$ (8)

where $\alpha_{0}=\sum_{j=1}^{3}\alpha_{j}$. The expectation of *E*(*θ*_*i*_|*D*) represents the probability of the stimulus *S*_*i*_ causing the outcome (causal strength), and the variance *Var*(*θ*_*i*_|*D*) represents the uncertainty of the causal estimate (causal uncertainty). The summation term α_0_ automatically accounts for latent inhibition, by which an increase in causal strength for a certain stimulus suppresses the causal strength of other stimuli. For example, given two stimuli, if α_1_ increases, then both the posterior mean for the first stimulus α_1_ / α_0_ and the value of the summation term α_0_ = α_1_ + α_2_ increase. This brings about a decrease in the posterior mean for the other stimulus α_2_ / α_0_.

#### Bayesian inference for causal learning: (3) Control of learning rate

To test our main hypothesis that the learning rate is controlled by means of the relative amount of causal uncertainty, we modified the Bayesian model in a way that the learning rate is determined by the relative amount of uncertainty in the causal relationship between each individual stimulus and an outcome. Our model converges as it resolves the uncertainty, whereas other types of associative models capable of learning rate control, including the Pearce-Hall [8] or McLaren model [9], converge after the same types of events repeatedly occur.

Given the amount of causal uncertainty for each individual stimulus, the model computes the learning rate according to the following softmax operation [46,75]. The learning rate for the *i*-th stimulus is given by

$$\gamma_{i}=\frac{\text{exp}(\tau Var(\theta_{i}|D))}{\sum_{j}\text{exp}(\tau Var(\theta_{j}|D))},$$ (9)

where *τ* is the inverse temperature parameter controlling the extent to which the model increases the learning rate for the stimulus with the higher posterior variance. In other words, the learning rate is proportional to the degree of relative causal uncertainty. The less the model’s posterior estimation about a stimulus is confident compared to other stimuli, the more rapidly the model learns about a stimulus-outcome association.

#### Bayesian inference for causal learning: (4) Novelty and modulation of salience

McLaren and Mackintosh’s formulation of associative learning [9] modifies the learning rate according to salience driven by cue novelty, whereby a learning rate is initially high in an early stage and then decreases as the same type of events repeatedly occurs. This promotes rapid learning for a novel stimulus and precludes an overshoot or oscillation as the learning progresses. This mechanism is also embedded in our Bayesian formulation (S11 Fig); the learning rate is high when the novelty of the stimulus is high, but it decreases as learning progresses.

#### Additional parameters—Primary and recency effect

When participants are asked to recall a list of stimuli after an outcome is presented, they tend to recall the first or the last item best. These cognitive biases are called the primacy and the recency effects, respectively [57,58]. To test these effects, we introduce two free parameters to each of the above models. The modified update rule is as follows:

$$\Delta \alpha_{i}=\gamma (x_{i}+\delta_{p}+\delta_{r}),$$ (10)

where $\delta_{p}=\{\begin{array}{cc} 1 & D(1)=S_{i}\\ 0 & otherwise \end{array}$ and $\delta_{r}=\{\begin{array}{cc} 1 & D(T)=S_{i}\\ 0 & otherwise \end{array}$, with *D*(*n*) being the *n*-th element of the event set *D*. The two free parameters *δ*_*p*_ and *δ*_*r*_ represent the extent to which the credit for the outcome goes to the first and the last item, respectively. The fitted parameter values of the best model are significantly greater than zero, indicating that they are neither trivial nor redundant. (The mean and the standard deviation of the primacy weight are 0.36 and 0.75, respectively; one-tailed one-sample *t* test; *p* = 0.0005. The mean and the standard deviation of the recency weight are 0.37 and 0.83, respectively; one-tailed one-sample *t* test; *p* = 0.001).

#### Model subtypes—Additive and sole effect

Another feature of how an agent might solve our task is that they may treat each individual stimulus encountered within a trial as separate or alternatively they may take the number of presentations of each stimulus into account, assuming that repetitive presentation strengthens or weakens the likelihood of outcome delivery (“additive effect”). For example, the amount of update of the causal strength when a stimulus is presented ten times before presentation of an outcome is ten times the amount of update when the stimulus is presented only once before the outcome presentation. The update rule described above, which counts the number of stimulus presentations, assumes that the additive effect exists.

We also tested an alternative version of each of the models, assuming that participants take the presence or absence of the stimulus into account when they update the causal strength (“sole effect”), as opposed to the number of presentations. For example, no matter how many times a stimulus is presented before an outcome presentation, the amount of update of the causal strength is the same as in the case in which the stimulus is presented once before the outcome presentation. When testing the version of the sole effect, *x*_*i*_ is 1 in the presence of the cue and 0 in the absence of the cue. To test these effects for each possible model type, we ran two different versions of each of the above causal learning models and fit each to the behavioral data. S1 Table illustrates how these effects were tested for each individual model.

#### Parameter estimation and model validation

The free parameters in these models are primacy/recency effect weights (described earlier), a baseline learning rate, and an inverse temperature of the softmax function. The baseline learning rate is used as a baseline for the modulation of the learning rate [9]; this parameter is also considered as a learning rate for the models incapable of learning rate modulation, i.e., the R-W, PCM, and Bayes model. The fourth parameter, the inverse temperature, determines the degree of relaxation for softmax operation [46].

We used the Nelder-Mead simplex algorithm [76] to estimate the parameters by minimizing the negative mean squared error of the rating ∑‖*E*(*θ*|*D*)−*R*‖^2^, summed over all trials for each subject, where *E*(*θ*|*D*) refers to the model’s causal strength vector and *R* refers to a subject’s normalized rating vector. The criterion reaches its minimum (zero) if the model’s causal strengths are equal to subjects’ normalized ratings in all trials. To minimize the risk of finding a local but not global optimal solution, we ran the optimization 100 times with randomly generated seed parameters. The optimal parameter values of each of the testing models are listed in S1 Table.

Nine versions of models were tested in total (each model detailed above implemented in sole and additive versions). S5A Fig shows the performance of each model in which the test model was chosen between the additive and sole effect version according to the model fit for each individual subject. The full model comparisons are shown in S5B Fig.

In order to compare performance of different models we used leave-one-out cross validation (LOOCV), which provides empirical evaluation of generalization performance of nonprobabilistic learning models [77]. It first creates a validation dataset using a single observation from the original dataset and a training dataset using the remaining observations and then repeats such that each observation in the sample is used once as the validation data.

#### Computer simulations—Empirical evaluation of model behavior (S1 Fig and S2 Fig)

In addition to the model comparison, we ran an extra computer simulation to empirically demonstrate that the model controls its learning rate in such a way that it reduces the total amount of causal uncertainty, thereby successfully converging in many different conditions. The simulation also shows event-by-event changes in learning rate. We performed model simulations of our BayesU model, the version that implements our computational hypothesis (refer to Materials and Methods for more details). The parameter values of the model are taken from a single model whose parameter values are the closest to the median of the parameter set of the models individually optimized for 47 subjects. This model can thus be considered as a prototype of the human inference process. In each simulation, stimuli and outcomes were randomly generated according to (i) the conditional probability distribution of an outcome given the stimulus: (1/2,1/2,1/2) or (1/16,1/8,1), and (ii) the ratio of frequency of stimulus presentation: (1:1:1) or (16:8:1). An uneven ratio refers to a case in which the degrees of frequency in which stimuli are presented differ. Each simulation is repeated 100 times. S1 Fig is intended (i) to substantiate the claim that the model minimizes the total amount of causal uncertainty in the relationship between a stimulus and an outcome and also (ii) to show under what conditions the model increases learning rates. S2 Fig further investigates the claim by demonstrating that the change in learning rate causes the reduction in total amount of causal uncertainty. We used the Granger-causality test, a statistical hypothesis test for determining whether one time series provides useful information to forecast another time series [78]. Specifically, we checked to see if the changes in learning rate can be used to predict the changes in total amount of causal uncertainty. Taken together, these simulations illustrate the role of learning rate control in resolving the uncertainty in causal relationship between a stimulus and an outcome.

## Supporting Information

S1 Data S1 Data provides anonymized behavioral data from the experiments plotted in Figs 3–6. (XLSX)

S2 Data S2 Data contains the SPM8 images of the group statistical maps for Fig 5A (familiarity). To view the images, SPM is needed (http://www.fil.ion.ucl.ac.uk/spm/software/spm8/).
(ZIP)

S3 Data S3 Data contains the SPM8 images of the group statistical maps for Fig 5A (causal uncertainty). To view the images, SPM is needed (http://www.fil.ion.ucl.ac.uk/spm/software/spm8/).
(ZIP)

S4 Data S4 Data contains the SPM8 images of the group statistical maps for Fig 5B (OS-IC). To view the images, SPM is needed (http://www.fil.ion.ucl.ac.uk/spm/software/spm8/).
(ZIP)

S1 Fig The learning process of the causal uncertainty model (BayesU) minimizes the total amount of uncertainty. Shown are the the causal strength (posterior mean) and causal uncertainty (posterior variance) over the course of learning (trial), as a result of computer simulations. Mean of posterior, a probability of a stimulus causing an outcome, refers to the causal strength, and the posterior variance refers to the causal uncertainty. The blue, green, and red lines correspond to the average posterior mean/variance for stimulus S1, S2, and S3, respectively. S1 and S2 refer to the non-novel cues, and S3 refers to a novel cue that is presented only once each round. The shaded areas are SEM across simulations. Each section (A–D) corresponds to the simulation in a different condition; (A) and (C) refer to a case in which the ratio of stimulus presentation frequency is 1:1:1, whereas (B) and (D) correspond to the ratio 16:8:1. (A) and (B) refer to a case in which the outcome probability given to each stimulus is (1/2,1/2,1/2), whereas (C) and (D) correspond to (1/16,1/8,1), respectively. In all of the cases A–D, the causal strength converged and the corresponding amount of causal uncertainty was minimized (the top and bottom left plots). In case A where the outcome probability and the frequency of stimuli presentation were balanced out, the learning rate remains constant around its baseline (the right plot of A; the baseline corresponds to the dotted orange line with each uncertainty amount being equal). In case B and C where either the outcome probability or the frequency of stimuli presentation were unbalanced, however, the learning rate deviates from its baseline (the right plot of B and C). The learning becomes more rapid when an outcome seems to be exclusively caused by a novel stimulus (case D); this is akin to the one-shot learning condition of our main experiment. This mechanism for modulating a learning rate (deviating from the baseline learning rate) seems to compensate for a lack of observations in minimizing the total amount of uncertainty. Refer to the Materials and Methods for full details of the computer simulation.
(TIF)

S2 Fig Balancing out control of learning rate serves to minimize the amount of total uncertainty. Shown are examples from the simulations in S1 Fig. The top figure in each subplot refers to the total amount of causal uncertainty (U), which is the sum of all the causal uncertainty for all the stimuli. The middle figure in each subplot refers to the trial-by-trial change of learning rate (L) for stimuli S1, S2, and S3. The learning rates are color coded; for example, the red and blue colors correspond to rapid and slow learning, respectively. The bottom figure in each subplot refers to the extent to which the learning rate for one stimulus is different from every other stimulus (variance of learning rate *Var*(*L*); orange line) and the amount of increase in the total amount of causal uncertainty (Δ*U* := *U*(*t* + 1) – *U*(*t*) with t being the trial index; green line); for example, the zero variance at the trial *t* (the orange line reaching the zero point) means that learning rates are equal for all stimuli at the trial *t*, and the zero Δ*U* at the trial *t* (zero-crossing of the green line) means that the total amount of causal uncertainty was not reduced compared to the trial *t*-1. When comparing the changes in the total amount of causal uncertainty (the top figure in each subplot) with the changes in individual learning rate (the middle figure in each subplot), the increase in learning rate seems to be negatively correlated with the total amount of uncertainty (the bottom figure in each subplot). Hence, to formally test if the learning rate control contributes to reduction of the total amount of uncertainty, we ran a Granger causality test [78] in which the causal effect of variance in learning rate (*Var*(*L*)) on changes in total amount of uncertainty (Δ*U*) is assessed. The test was repeated 100 times for each condition. We found a significant causal effect; variance in learning rate causes the reduction in total amount of uncertainty (Granger causality test: F = 768.2, critical value = 4.2, alpha = 1e-5; correlation coefficient = -0.45, *p* < 1e-5; significant at the 0.001% significance level), indicating that the model indeed controls the learning rate in such a way that it reduces the total amount of causal uncertainty. Refer to Materials and Methods for full details of the computer simulation.
(TIF)

S3 Fig Relationship between the learning rate threshold and incremental/one-shot learning. (A) Shown are the histograms of learning rate (the frequency distribution of the learning rates) for each individual subject who was scanned with fMRI. To divide the events into two types according to learning rate, we fit a Gaussian mixture model using an expectation maximization algorithm [79]. Two distributions, respectively representing slow or rapid learning, were found in 17 out of 20 subjects. The center of each cluster is indicated by a green circle. The green dotted line indicates a boundary between the two distributions, for which the two adjacent learning rate points have the same posterior probability. The red dotted line indicates the 90th percentile of learning rate. (B) The scatter plot (left) shows that the boundary between the two distributions is very close to the 90th percentile learning rate value. The coefficient of determination is 0.86, indicating that the 90th percentile learning rate fits well the boundary between the slow and the rapid learning process predicted by the Gaussian mixture model. The bar plot (right) also shows there is no significant difference between the 90th percentile and the boundary value (paired-sample *t* test; *p* = 0.5). Seventeen out of 20 subjects’ data (*n* = 17) were used for these analyses after we excluded three subjects for which only one distribution was found. These results indicate that the 90th percentile threshold is a viable predictor for distinguishing between one-shot and incremental learning.
(TIF)

S4 Fig Occurrence of one-shot learning events across non-novel and novel stimuli. Related to Fig 4B. Shown on the left is the proportion of three subtypes of rounds separated according to the causal uncertainty model’s prediction (IC round/ OS familiar/ OS novel) and as a function of stimulus and outcome novelty (type1 versus type2 round). IC round refers to the rounds during which the model predicts no occurrence of one-shot learning events, and OS familiar and OS novel refer to the rounds during which the model predicts that one-shot learning events occur at the time of presentation of non-novel stimuli and a novel stimulus, respectively. Shown in the right are the dominant patterns of a sequence of events for each subtype of rounds (IC round/ OS familiar/ OS novel). Specifically, we conducted principal component analysis on data vectors generated by concatenating sequences of stimulus/outcome events and computed the first component of a data vector that loads the greatest variance. In the Type1 round, the amount of causal uncertainty for non-novel stimuli decreases as they are repeatedly paired with a non-novel outcome (trials 1–3). However, the amount of causal uncertainty for non-novel stimuli increases after they are unexpectedly paired with a novel outcome, resulting in an increase in the corresponding learning rate at the end of this trial (trial 4 of both the OS familiar and the OS novel rounds). In 20% of the type1 rounds, the increased amount of causal uncertainty for one of the non-novel stimuli is greater than that for a novel stimulus, resulting in a high learning rate assigned to the non-novel stimulus (trial 5 of the OS familiar rounds), whereas in 60% of the type1 rounds the amount is still less than that for the novel stimulus, resulting in a high learning rate assigned to the novel stimulus (trial 5 of the OS novel rounds). Each row represents each trial; the first trials are omitted for simplicity. The stimulus/outcome identity is color coded. The most frequently presented stimulus (presented 16 out of 25 times), the second most frequently presented one (eight out of 25 times), and the novel stimulus (one out of 25 times) are coded as blue, yellow, and red, respectively; for example, the blue and red cells indicate that there is a high chance of a non-novel or novel stimulus/outcome presentation, respectively. The bold black circle in a cell indicates that the model predicts the occurrence of one-shot learning during that event; for example, the circles in the blue and orange cells correspond to the occurrence of one-shot learning as predicted for a non-novel and a novel stimulus presentation, indicating that one-shot learning is not confined to a novel stimulus. Taken together, this illustrates how one-shot learning (relatively large amount of causal uncertainty) arises for non-novel and novel stimuli within each subtype of rounds.
(TIF)

S5 Fig Performance comparison. Leave-one-out cross validation was used to validate the generalization performance of the models. R-W refers to the Rescorla-Wagner model [6], PCM refers to Jenkin’s probabilistic contrast model [7], and P-H model refers to the Pearce-Hall model [8]. Bayes refers to a Bayesian causal model (see Materials and Methods), and BayesU refers to the causal uncertainty model proposed in the present study, which is a variant of a Bayesian causal learning model in which the learning rate is controlled by relative causal uncertainty (see Materials and Methods). BayesM is the same as BayesU, except that the learning rate is determined based on causal strength. Random model refers to a model that generates random ratings, BayesStruct refers to Bayesian causal structure learning [48,74], and Heuristic refers to a post hoc model of heuristic causal judgments. The models were fitted to each individual subject’s data. (A) The green bar (“sole-effect version”) refers to the case in which we tested the sole-effect version of the model, in which the presence or absence of the stimulus is assumed to be taken into account, and the orange bar (“additive-effect version”) refers to the case in which we tested the additive effect, assuming that repetitive presentation strengthens or weakens the likelihood of an outcome. The white bar (“winner version”) refers to the case in which the test models were chosen between the additive and sole-effect version according to the model fit for each individual subject. The model performance of BayesU model (the winner version) was significantly better than all the other models (Paired-sample *t* test; *p* < 0.01), confirming our hypothesis that the learning rate is determined based on relative causal uncertainty. The estimated parameters are listed in S1 Table. (B) Summary of the model comparison. Tested were the “winner versions” in the analysis (A) and the three nonparametric models: Random, BayesStruct, and Heuristic. The performance of BayesU is significantly better than all the other models (Paired-sample *t* test; *: *p* < 0.05, **: *p* < 0.01). Error bars are SEM across subjects.
(TIF)

S6 Fig Models’ ratings and one-shot effect, categorized by round types. The results from the best-fitting versions of each model (S5 Fig) are used for display. The type1 round and the type2 round refer to rounds in which a novel cue is paired with a non-novel outcome and with a novel outcome, respectively. The first row shows the subjects’ ratings and the corresponding one-shot effect indices, and the second and the third row show the models’ ratings and the one-shot effect indices. The one-shot effect index is defined as the rating for the novel cue minus the average rating for the non-novel cues. Rescorla-Wagner (R-W), probabilistic contrast model (PCM), Heuristic, BayesM, and BayesU show a one-shot learning effect that is qualitatively similar to that shown by subjects. However, BayesU is the only model that exhibits the closest rating patterns to the patterns of subjects in terms of the one-shot effect index, which is independent of the criterion used for the model optimization (mean squared error). Note that the average rating patterns shown here reflect only the main effect of the causal learning task. The results are corroborated by the formal model comparison (S5 Fig). SEM across subjects and rounds are shown as error bars for display purposes.
(TIF)

S7 Fig Neural activity in subregions of hippocampus increases only if the learning rate is very high. We used anatomically defined hippocampus ROIs [54]: CA1–CA3 (cornu ammonis [CA]), dentate gyrus (DG), and hippocampal-amygdala transition area (HATA). The neural activity in many parts of hippocampus increases significantly in OS but not in IC (paired-sample *t* test *p* < 1e-3). Error bars are SEM.
(TIF)

S8 Fig Summary of imaging results shown in Fig 5A and Fig 5B, using an analysis in which serial orthogonalization is disabled. All of the findings reported in the main analysis are preserved without orthogonalization at the same corrected thresholds used in the main analysis. These results indicate that our results are not an artifact of the orthogonalization approach. The color bars display *t* scores.
(TIF)

S9 Fig Sifting the evidence for task structure learning—Behavioral analysis. To check if participants learned a task structure insofar as expectations based on learned structure affected our results, we quantified the effect of structure learning on causal ratings. (A) We first checked if participants’ causal ratings exhibited one-shot learning effects in very early stages of learning, in which there is little chance of developing structure learning. The one-shot effect index patterns for the early rounds are the same as the patterns for the rest of the rounds (paired-sample *t* test; *p* > 0.1). *: *p* < 1e-2; one-sample *t* test. (B) We further tested to see if participants gradually developed structure learning over the course of rounds, i.e., if there is a linear dependence between the one-shot learning effect and rounds (e.g., enhanced or reduced one-shot learning effect over time). We carried out a regression analysis by fitting the first order model to each subjects’ data (explanatory variable: round number, dependent variable: one-shot effect index). Nonsignificant regression coefficients mean that there is no measurable effect of structure learning on causal ratings. Error bars are SEM across subjects.
(TIF)

S10 Fig Sifting the evidence for task structure learning—Model-based analysis. Related to S9 Fig. To quantify statistical regularity in causal associations as would be predicted by a structure-learning effect, we fit the Bayesian causal structure learning model to participants’ causal rating data to infer underlying causal associations and then conducted a principal component analysis on data vectors of the causal associations, followed by quantification of the amount of variance explained by the dominant patterns of causal association. If structure learning is the major factor that affects causal ratings, the dominant patterns of causal association should explain most of the variance. The strong structure learning hypothesis, indicated by the red dotted lines, refers to the case in which it is assumed that the respective learned task structure for the type1 and type2 round is used to make predictions about causal ratings. It is implemented by fitting the Bayesian causal structure learning model to the causal rating data of the type1 and type2 round, respectively, followed by adding 10% noise to its predictions. The weak structure learning hypothesis, indicated by the orange dotted lines, is the same as the strong structure learning one, except for adding 50% noise to its predictions. The subjects’ causal associations, indicated by the grey box plot, refer to the case in which the Bayesian causal structure learning model was fit to each individual round’s data to infer underlying causal associations. The random causal associations, indicated by the black dotted lines, refer to the case in which the causal associations are randomly generated from a uniform distribution. Shown are the proportions of explained variance (A: normalized eigenvalues for the top three eigenvectors; B: for the first eigenvector only) in causal associations predicted by each case. We found that the most dominant patterns of causal associations predicted by the subject’s causal associations model explain only 32% of variance, which is significantly less than the weak structure learning effect (45%) yet significantly greater than the random causal associations (13%) effect (paired-sample *t* test on explained variables; *p* < 0.001), as shown in the normalized eigenvalues for the first eigenvectors, suggesting that there is no measurable regularity in causal association patterns.
(TIF)

S11 Fig Effect of the number of cue occurrences on the posterior estimation. Shown are the posterior mean and variance of the Bayesian causal inference model as a function of the total number of cue occurrence. The model considers two cues and one outcome. The ratio m:n means that the ratio of the occurrence of the first cue and the second cue is m:n. For example, the point of the red line (10,0.83) in the left figure refers to the case in which the first cue was presented nine times out of ten, the second cue was presented one time, and the model’s estimate of the posterior mean was 0.83. There is a rapid update in the early phase of learning and then the update becomes slower as learning progresses, which adequately reflects the modulation of learning rate based on novelty of stimuli [9].
(TIF)

S1 Table Computational models for causal inference. List of the models and estimated parameter values. Random: model generating a random causal rating, Heuristic: model of heuristic causal judgment that learns from novel stimulus-novel outcome association, R-W: delta-rule model based on the Rescorla-Wagner model [6], PCM: probabilistic contrast model (Jenkins and Ward, 1965), P-H: Pearce-Hall model [8], BayesStruct: online Monte Carlo Markov Chain method for learning causal Bayesian network structures [48,74], Bayes: Bayesian inference of causal learning, BayesM: a variant of the Bayes model [47] in which the learning rate is determined based on causal strength, BayesU: a variant of the Bayes model [47] in which the learning rate is determined based on causal uncertainty. “O” indicates that the model has been created and tested. “N/A” means the model is not suitable for that type of test. Model subtypes: additive effect refers to the version of models assuming that repetitive presentation strengthens or weakens the likelihood of outcome delivery, and sole effect refers to the version of models assuming that participants take the presence or absence of the stimulus into account when they update the causal strength. Winner refers to which of the model versions was chosen for each participant. A comparison between the sole effect and the additive effect is shown in S5A Fig, and the full model comparison for the winner version is shown in S5B Fig. Note that for model comparison we used leave-one-out cross validation (LOOCV), which provides empirical evaluation of generalization performance of nonprobabilistic learning models [77]. The LOOCV thus takes model complexity into account. Between-subjects mean and standard deviation, 47 subjects; parenthesis: standard deviation.
(TIF)

S2 Table Neural signatures of causal learning system. p(FWE): corresponds to peak level if the z-score indicates * and corresponds to cluster level if the z-score indicates +. vlPFC, ventrolateral prefrontal cortex; dmPFC, dorsomedial prefrontal cortex; dlPFC, dorsolateral prefrontal cortex; iOFC, inferior orbitofrontal cortex; IPL, inferior parietal lobule; MTG, middle temporal gyrus; ITL, inferior temporal lobe; FFG, fusiform gyrus. All the areas (marked with either “+” or “*”) survived after the-brain correction for multiple comparison at the cluster level (corresponding to “+”; height threshold *t* = 3.53, extent > 100 voxels).
(TIF)

## Abbreviations

AC-PC: anterior commissure-posterior commissure
BOLD: blood-oxygen-level dependent
CA: cornu ammonis
DG: dentate gyrus
dlPFC: dorsolateral prefrontal cortex
dmPFC: dorsomedial prefrontal cortex
EPI: echo-planar imaging
FFG: fusiform gyrus
fMRI: functional magnetic resonance imaging
FWE: family-wise error
GLM: general linear model
HATA: hippocampal-amygdala transition area
IC: incremental learning event
IC round: incremental learning round
iOFC: inferior orbitofrontal cortex
IPL: inferior parietal lobule
ITL: inferior temporal lobe
LOOCV: leave-one-out cross validation
MTG: middle temporal gyrus
OS: one-shot learning event
OS round: one-shot learning round
PCM: probabilistic contrast model
ROI: region of interest
R-W: Rescorla-Wagner model
SEM: standard error of the mean
SPM: statistical parametric mapping
vIPFC: ventrolateral prefrontal cortex
