# Supplementary figures and images for: Neural Computations Mediating One-Shot Learning in the Human Brain
Source: PLoS Biol. 2015 Apr 28;13(4):e1002137. doi: 10.1371/journal.pbio.1002137 (PMC4412411; doi:10.1371/journal.pbio.1002137)

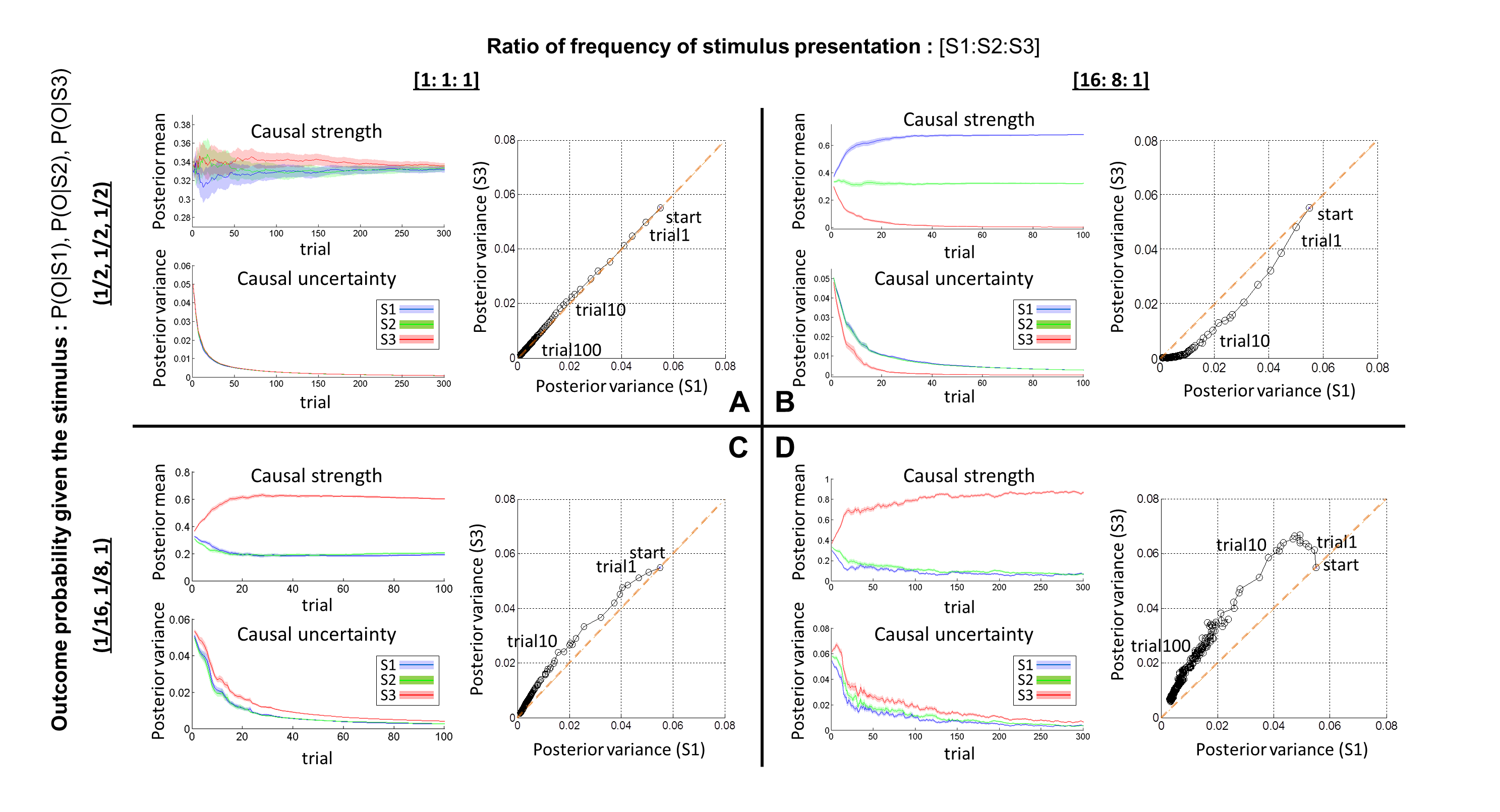

Supplement: S1 Fig — Shown are the the causal strength (posterior mean) and causal uncertainty (posterior variance) over the course of learning (trial), as a result of computer simulations. Mean of posterior, a probability of a stimulus causing an outcome, refers to the causal strength, and the posterior variance refers to the causal uncertainty. The blue, green, and red lines correspond to the average posterior mean/variance for stimulus S1, S2, and S3, respectively. S1 and S2 refer to the non-novel cues, and S3 refers to a novel cue that is presented only once each round. The shaded areas are SEM across simulations. Each section (A–D) corresponds to the simulation in a different condition; (A) and (C) refer to a case in which the ratio of stimulus presentation frequency is 1:1:1, whereas (B) and (D) correspond to the ratio 16:8:1. (A) and (B) refer to a case in which the outcome probability given to each stimulus is (1/2,1/2,1/2), whereas (C) and (D) correspond to (1/16,1/8,1), respectively. In all of the cases A–D, the causal strength converged and the corresponding amount of causal uncertainty was minimized (the top and bottom left plots). In case A where the outcome probability and the frequency of stimuli presentation were balanced out, the learning rate remains constant around its baseline (the right plot of A; the baseline corresponds to the dotted orange line with each uncertainty amount being equal). In case B and C where either the outcome probability or the frequency of stimuli presentation were unbalanced, however, the learning rate deviates from its baseline (the right plot of B and C). The learning becomes more rapid when an outcome seems to be exclusively caused by a novel stimulus (case D); this is akin to the one-shot learning condition of our main experiment. This mechanism for modulating a learning rate (deviating from the baseline learning rate) seems to compensate for a lack of observations in minimizing the total amount of uncertainty. Refer to the Materials and M [file pbio.1002137.s005.tif]

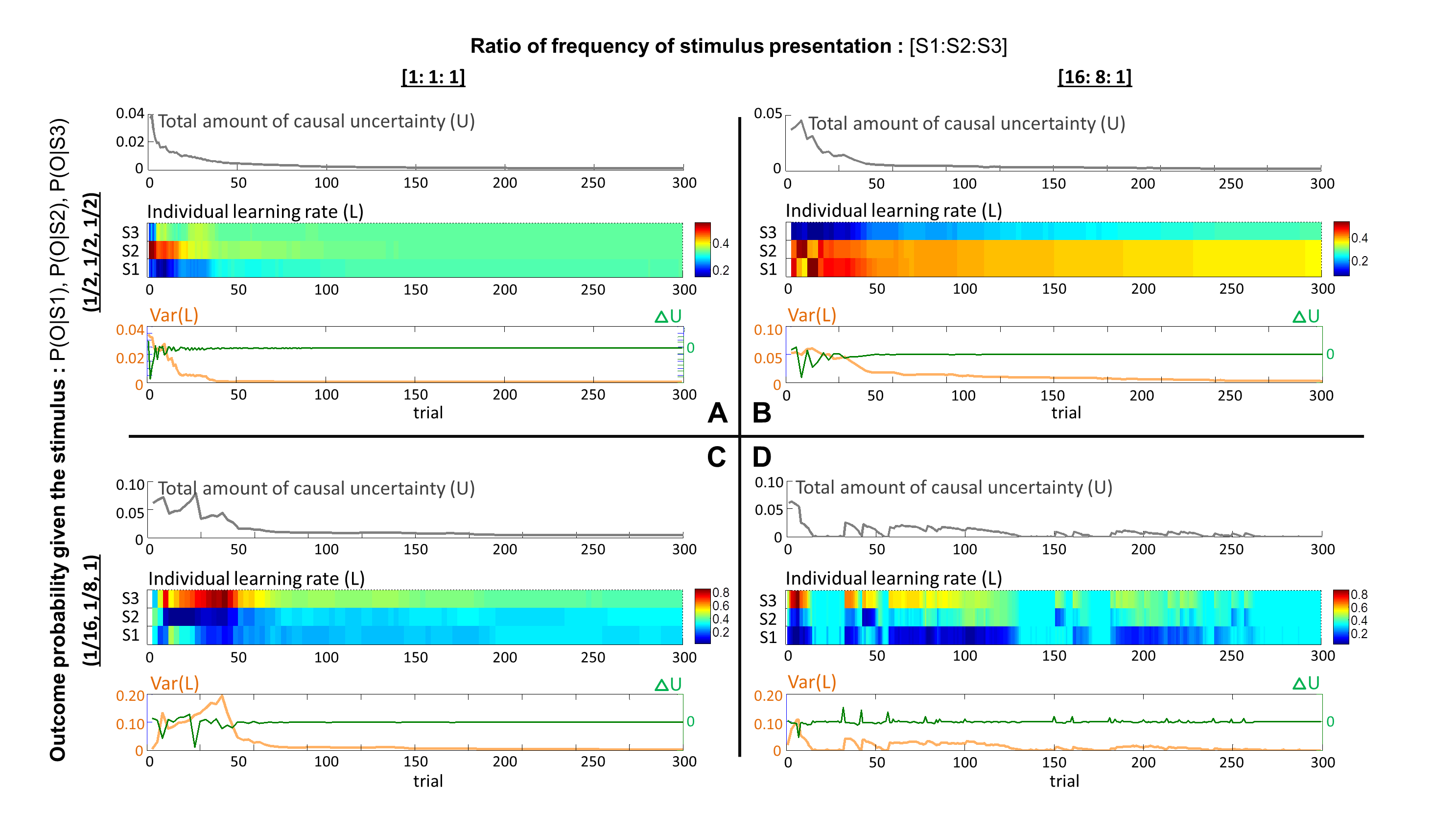

Supplement: S2 Fig — Shown are examples from the simulations in S1 Fig. The top figure in each subplot refers to the total amount of causal uncertainty (U), which is the sum of all the causal uncertainty for all the stimuli. The middle figure in each subplot refers to the trial-by-trial change of learning rate (L) for stimuli S1, S2, and S3. The learning rates are color coded; for example, the red and blue colors correspond to rapid and slow learning, respectively. The bottom figure in each subplot refers to the extent to which the learning rate for one stimulus is different from every other stimulus (variance of learning rate Var(L); orange line) and the amount of increase in the total amount of causal uncertainty (ΔU := U(t + 1) – U(t) with t being the trial index; green line); for example, the zero variance at the trial t (the orange line reaching the zero point) means that learning rates are equal for all stimuli at the trial t, and the zero ΔU at the trial t (zero-crossing of the green line) means that the total amount of causal uncertainty was not reduced compared to the trial t-1. When comparing the changes in the total amount of causal uncertainty (the top figure in each subplot) with the changes in individual learning rate (the middle figure in each subplot), the increase in learning rate seems to be negatively correlated with the total amount of uncertainty (the bottom figure in each subplot). Hence, to formally test if the learning rate control contributes to reduction of the total amount of uncertainty, we ran a Granger causality test [78] in which the causal effect of variance in learning rate (Var(L)) on changes in total amount of uncertainty (ΔU) is assessed. The test was repeated 100 times for each condition. We found a significant causal effect; variance in learning rate causes the reduction in total amount of uncertainty (Granger causality test: F = 768.2, critical value = 4.2, alpha = 1e-5; correlation coefficient = -0.45, p < 1e-5; significant at the 0.001% significa [file pbio.1002137.s006.tif]

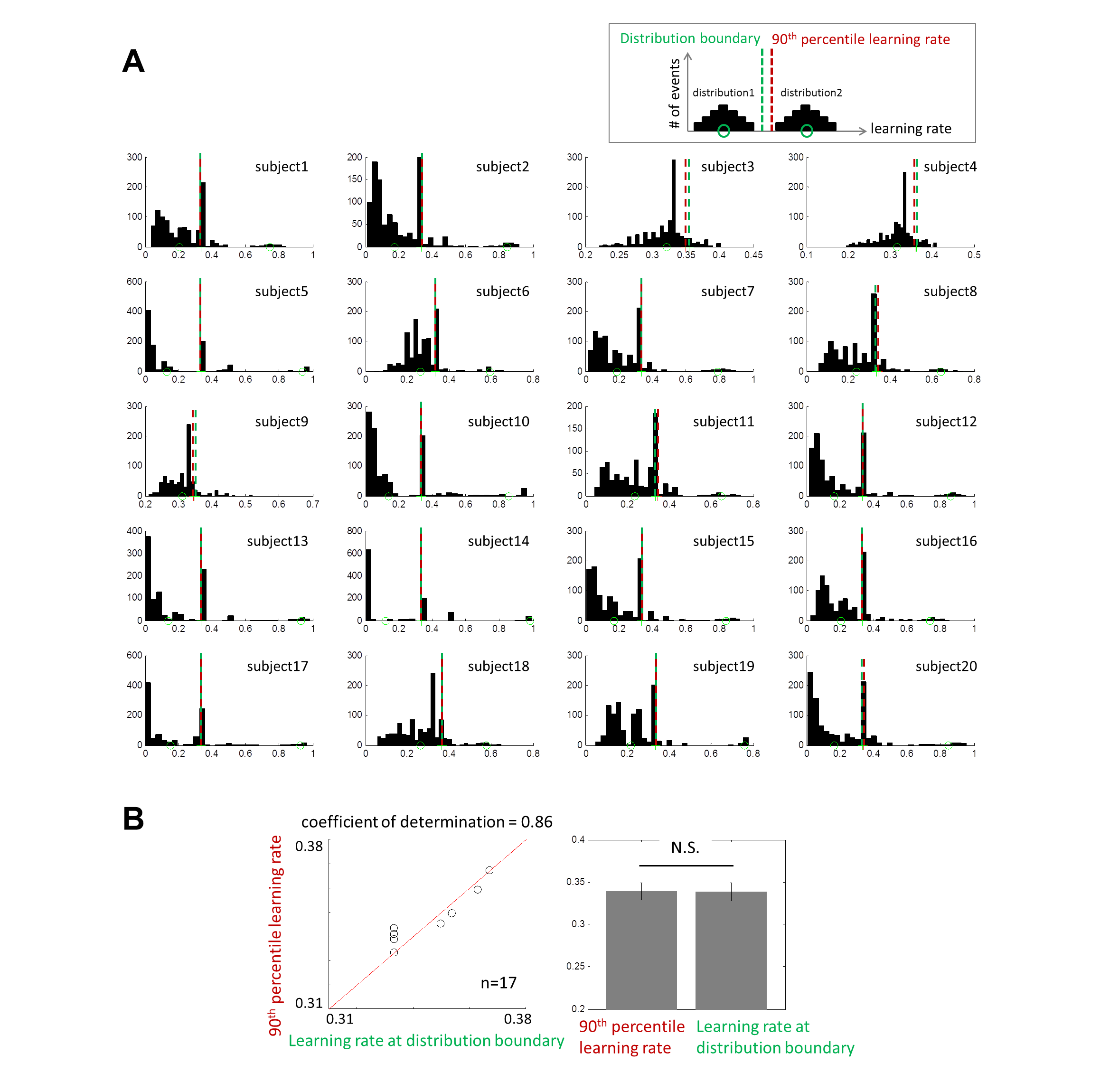

Supplement: S3 Fig — (A) Shown are the histograms of learning rate (the frequency distribution of the learning rates) for each individual subject who was scanned with fMRI. To divide the events into two types according to learning rate, we fit a Gaussian mixture model using an expectation maximization algorithm [79]. Two distributions, respectively representing slow or rapid learning, were found in 17 out of 20 subjects. The center of each cluster is indicated by a green circle. The green dotted line indicates a boundary between the two distributions, for which the two adjacent learning rate points have the same posterior probability. The red dotted line indicates the 90th percentile of learning rate. (B) The scatter plot (left) shows that the boundary between the two distributions is very close to the 90th percentile learning rate value. The coefficient of determination is 0.86, indicating that the 90th percentile learning rate fits well the boundary between the slow and the rapid learning process predicted by the Gaussian mixture model. The bar plot (right) also shows there is no significant difference between the 90th percentile and the boundary value (paired-sample t test; p = 0.5). Seventeen out of 20 subjects’ data (n = 17) were used for these analyses after we excluded three subjects for which only one distribution was found. These results indicate that the 90th percentile threshold is a viable predictor for distinguishing between one-shot and incremental learning. (TIF) [file pbio.1002137.s007.tif]

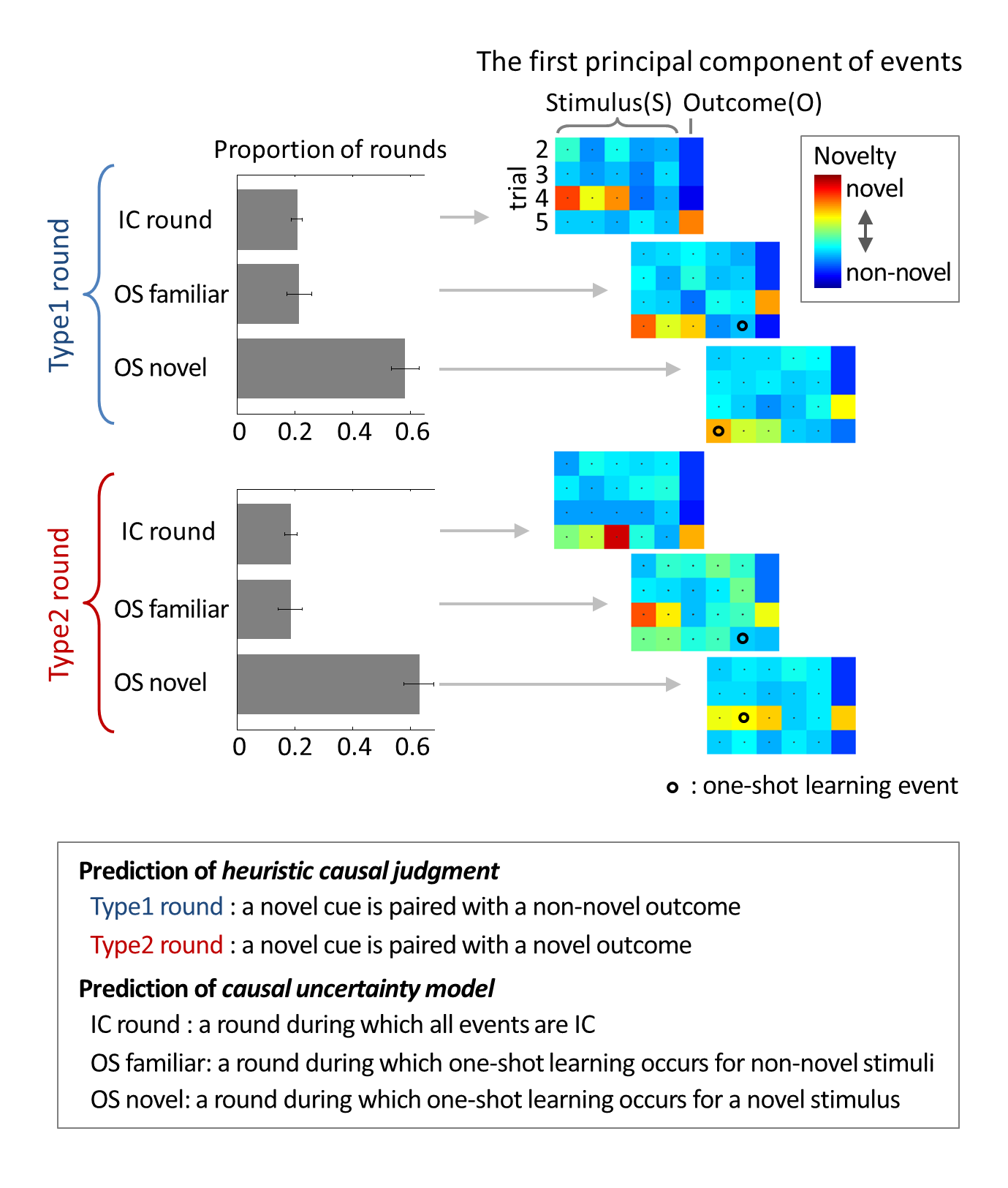

Supplement: S4 Fig — Related to Fig 4B. Shown on the left is the proportion of three subtypes of rounds separated according to the causal uncertainty model’s prediction (IC round/ OS familiar/ OS novel) and as a function of stimulus and outcome novelty (type1 versus type2 round). IC round refers to the rounds during which the model predicts no occurrence of one-shot learning events, and OS familiar and OS novel refer to the rounds during which the model predicts that one-shot learning events occur at the time of presentation of non-novel stimuli and a novel stimulus, respectively. Shown in the right are the dominant patterns of a sequence of events for each subtype of rounds (IC round/ OS familiar/ OS novel). Specifically, we conducted principal component analysis on data vectors generated by concatenating sequences of stimulus/outcome events and computed the first component of a data vector that loads the greatest variance. In the Type1 round, the amount of causal uncertainty for non-novel stimuli decreases as they are repeatedly paired with a non-novel outcome (trials 1–3). However, the amount of causal uncertainty for non-novel stimuli increases after they are unexpectedly paired with a novel outcome, resulting in an increase in the corresponding learning rate at the end of this trial (trial 4 of both the OS familiar and the OS novel rounds). In 20% of the type1 rounds, the increased amount of causal uncertainty for one of the non-novel stimuli is greater than that for a novel stimulus, resulting in a high learning rate assigned to the non-novel stimulus (trial 5 of the OS familiar rounds), whereas in 60% of the type1 rounds the amount is still less than that for the novel stimulus, resulting in a high learning rate assigned to the novel stimulus (trial 5 of the OS novel rounds). Each row represents each trial; the first trials are omitted for simplicity. The stimulus/outcome identity is color coded. The most frequently presented stimulus (presented 16 out of 25 times), the second mo [file pbio.1002137.s008.tif]

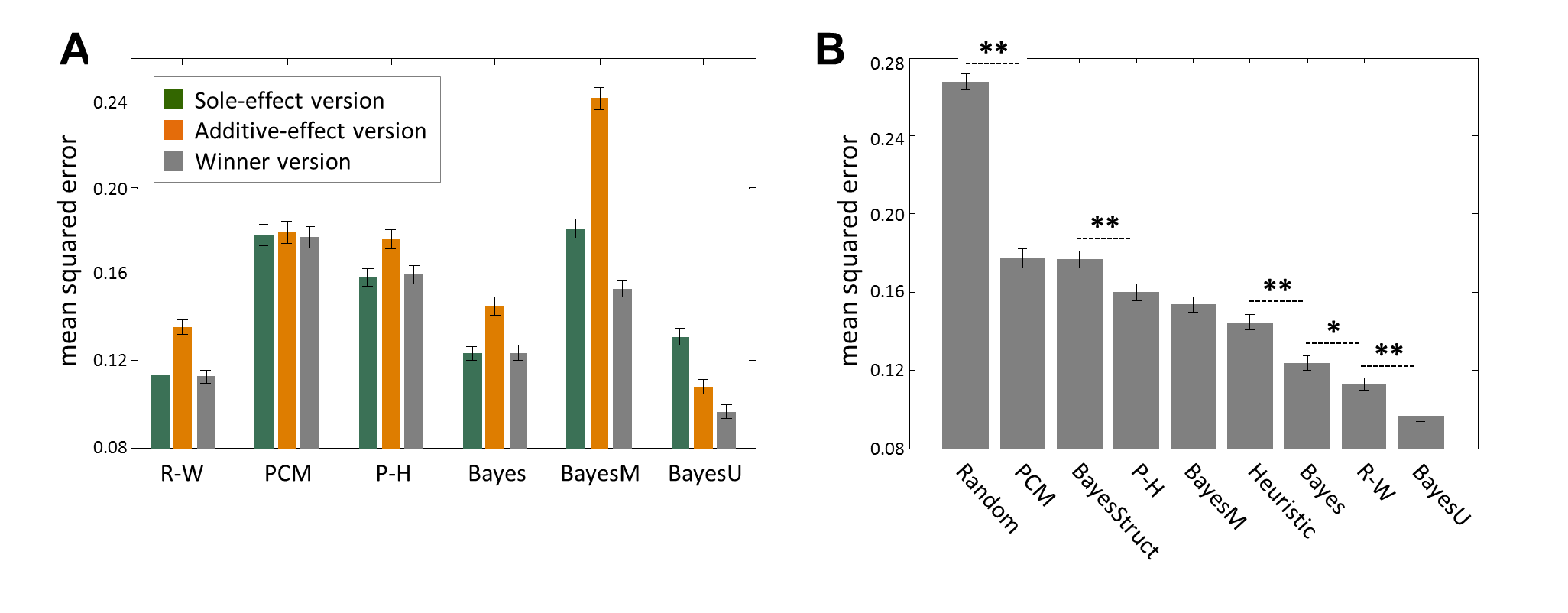

Supplement: S5 Fig — Leave-one-out cross validation was used to validate the generalization performance of the models. R-W refers to the Rescorla-Wagner model [6], PCM refers to Jenkin’s probabilistic contrast model [7], and P-H model refers to the Pearce-Hall model [8]. Bayes refers to a Bayesian causal model (see Materials and Methods), and BayesU refers to the causal uncertainty model proposed in the present study, which is a variant of a Bayesian causal learning model in which the learning rate is controlled by relative causal uncertainty (see Materials and Methods). BayesM is the same as BayesU, except that the learning rate is determined based on causal strength. Random model refers to a model that generates random ratings, BayesStruct refers to Bayesian causal structure learning [48,74], and Heuristic refers to a post hoc model of heuristic causal judgments. The models were fitted to each individual subject’s data. (A) The green bar (“sole-effect version”) refers to the case in which we tested the sole-effect version of the model, in which the presence or absence of the stimulus is assumed to be taken into account, and the orange bar (“additive-effect version”) refers to the case in which we tested the additive effect, assuming that repetitive presentation strengthens or weakens the likelihood of an outcome. The white bar (“winner version”) refers to the case in which the test models were chosen between the additive and sole-effect version according to the model fit for each individual subject. The model performance of BayesU model (the winner version) was significantly better than all the other models (Paired-sample t test; p < 0.01), confirming our hypothesis that the learning rate is determined based on relative causal uncertainty. The estimated parameters are listed in S1 Table. (B) Summary of the model comparison. Tested were the “winner versions” in the analysis (A) and the three nonparametric models: Random, BayesStruct, and Heuristic. The performance of BayesU is signific [file pbio.1002137.s009.tif]

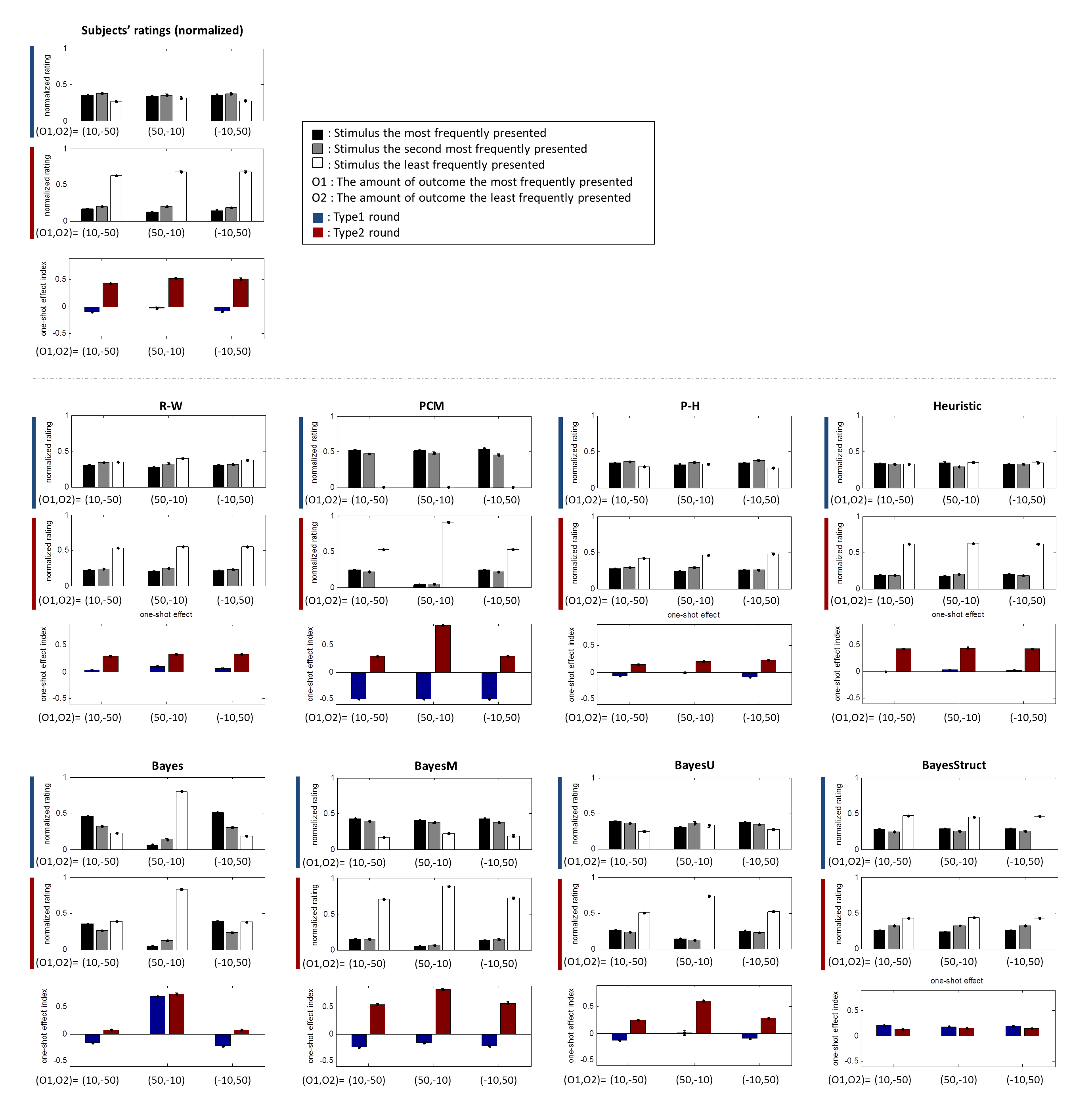

Supplement: S6 Fig — The results from the best-fitting versions of each model (S5 Fig) are used for display. The type1 round and the type2 round refer to rounds in which a novel cue is paired with a non-novel outcome and with a novel outcome, respectively. The first row shows the subjects’ ratings and the corresponding one-shot effect indices, and the second and the third row show the models’ ratings and the one-shot effect indices. The one-shot effect index is defined as the rating for the novel cue minus the average rating for the non-novel cues. Rescorla-Wagner (R-W), probabilistic contrast model (PCM), Heuristic, BayesM, and BayesU show a one-shot learning effect that is qualitatively similar to that shown by subjects. However, BayesU is the only model that exhibits the closest rating patterns to the patterns of subjects in terms of the one-shot effect index, which is independent of the criterion used for the model optimization (mean squared error). Note that the average rating patterns shown here reflect only the main effect of the causal learning task. The results are corroborated by the formal model comparison (S5 Fig). SEM across subjects and rounds are shown as error bars for display purposes. (TIF) [file pbio.1002137.s010.tif]

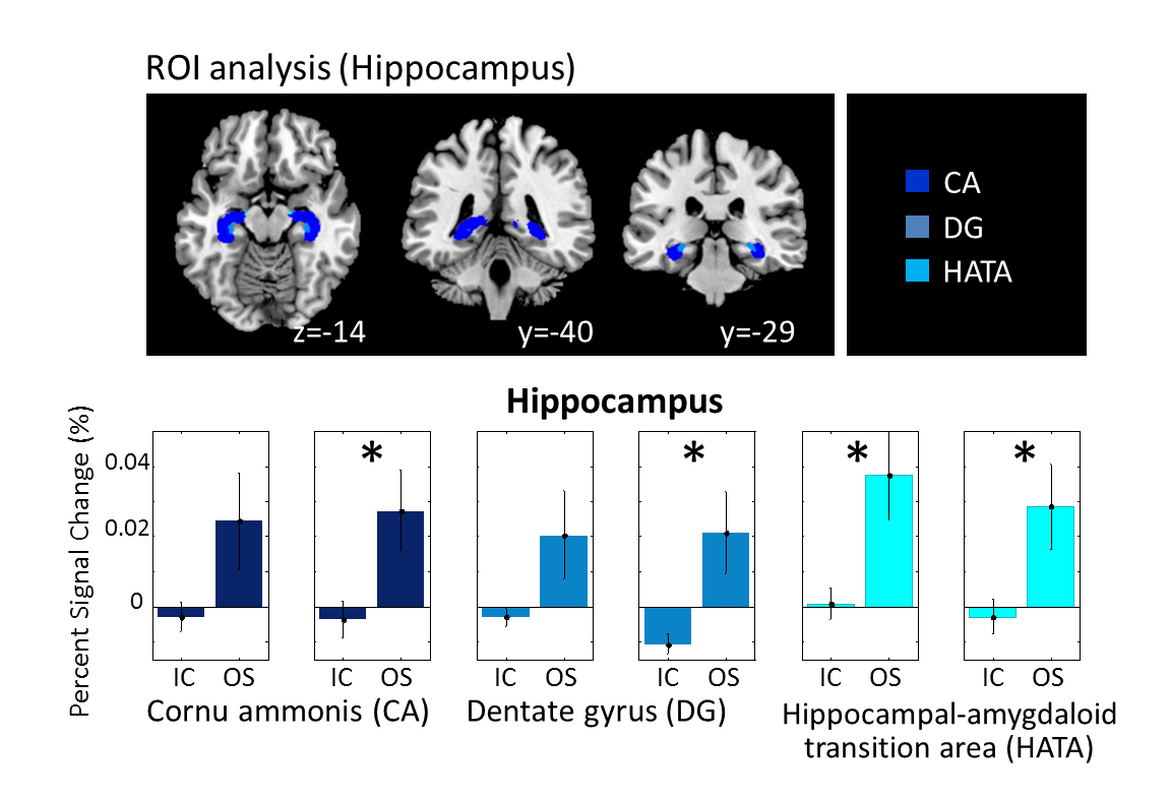

Supplement: S7 Fig — We used anatomically defined hippocampus ROIs [54]: CA1–CA3 (cornu ammonis [CA]), dentate gyrus (DG), and hippocampal-amygdala transition area (HATA). The neural activity in many parts of hippocampus increases significantly in OS but not in IC (paired-sample t test p < 1e-3). Error bars are SEM. (TIF) [file pbio.1002137.s011.tif]

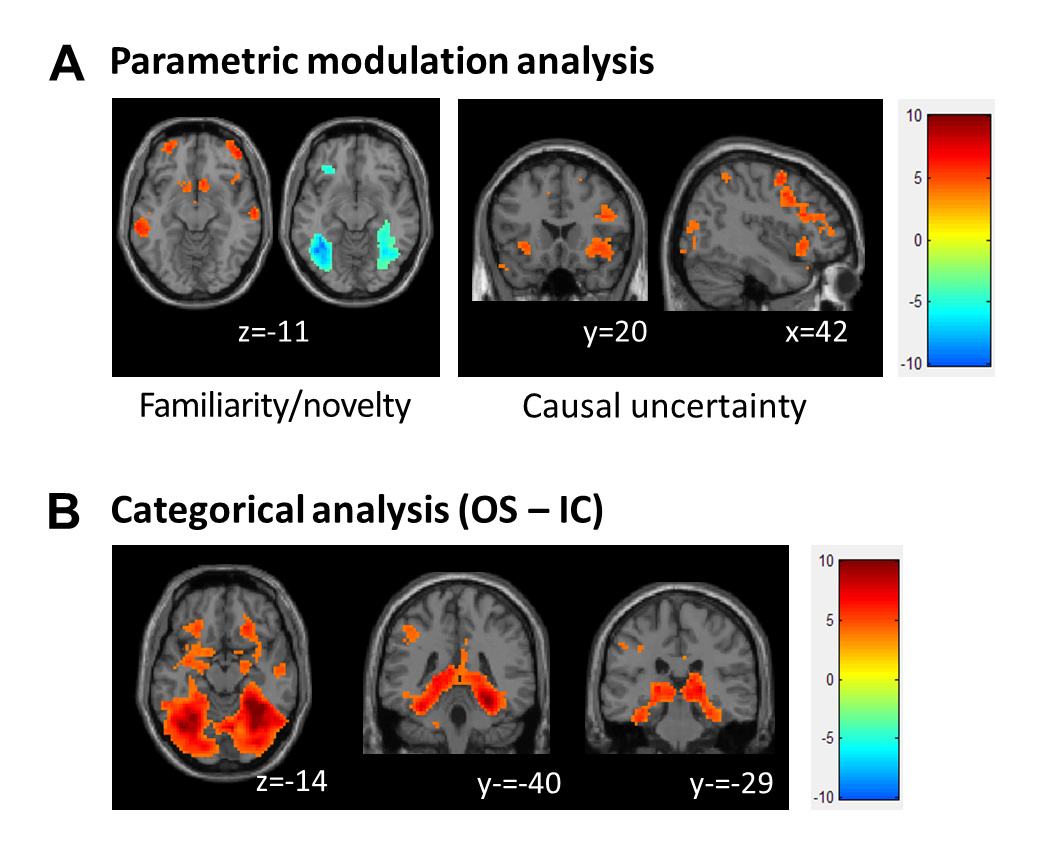

Supplement: S8 Fig — All of the findings reported in the main analysis are preserved without orthogonalization at the same corrected thresholds used in the main analysis. These results indicate that our results are not an artifact of the orthogonalization approach. The color bars display t scores. (TIF) [file pbio.1002137.s012.tif]

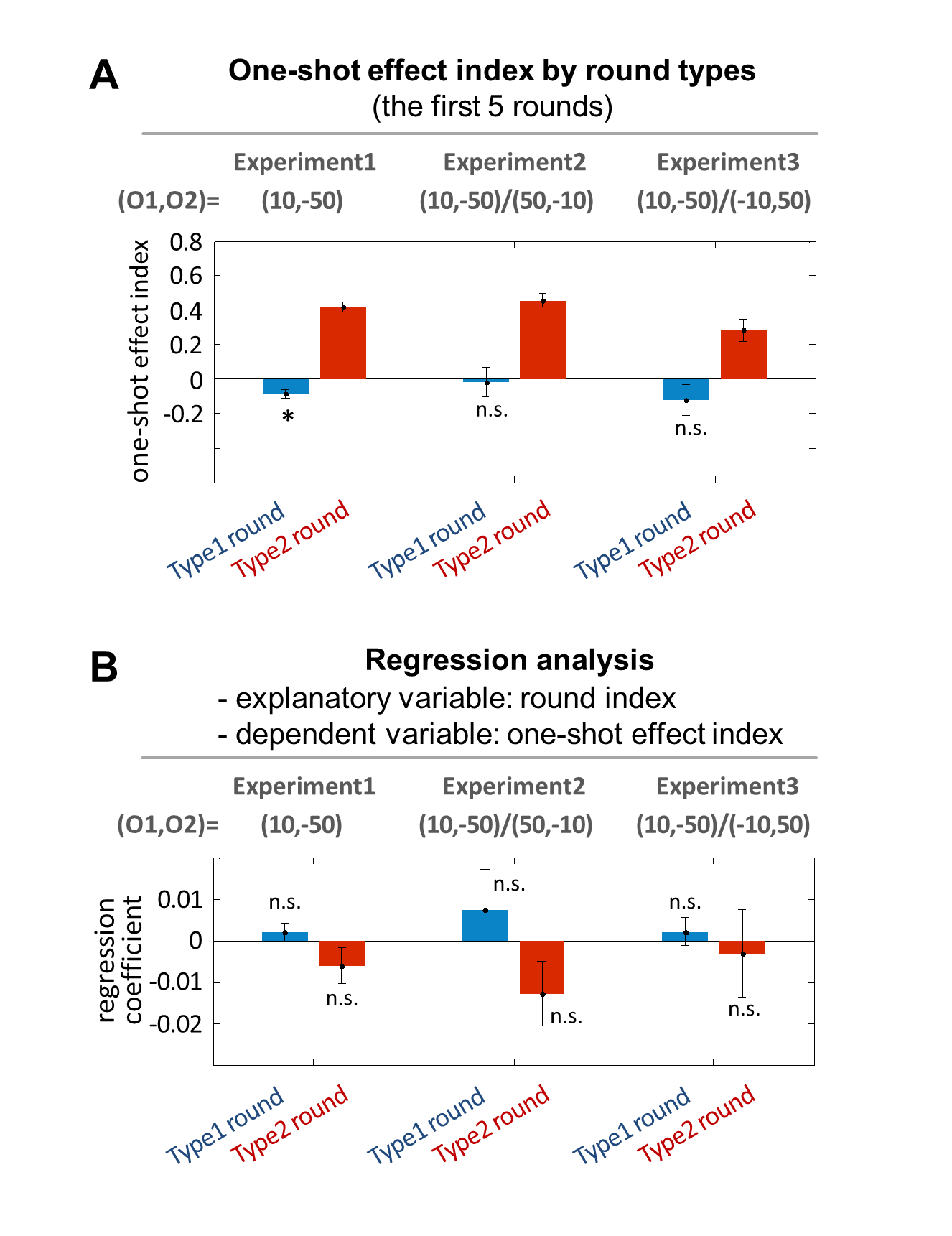

Supplement: S9 Fig — To check if participants learned a task structure insofar as expectations based on learned structure affected our results, we quantified the effect of structure learning on causal ratings. (A) We first checked if participants’ causal ratings exhibited one-shot learning effects in very early stages of learning, in which there is little chance of developing structure learning. The one-shot effect index patterns for the early rounds are the same as the patterns for the rest of the rounds (paired-sample t test; p > 0.1). *: p < 1e-2; one-sample t test. (B) We further tested to see if participants gradually developed structure learning over the course of rounds, i.e., if there is a linear dependence between the one-shot learning effect and rounds (e.g., enhanced or reduced one-shot learning effect over time). We carried out a regression analysis by fitting the first order model to each subjects’ data (explanatory variable: round number, dependent variable: one-shot effect index). Nonsignificant regression coefficients mean that there is no measurable effect of structure learning on causal ratings. Error bars are SEM across subjects. (TIF) [file pbio.1002137.s013.tif]

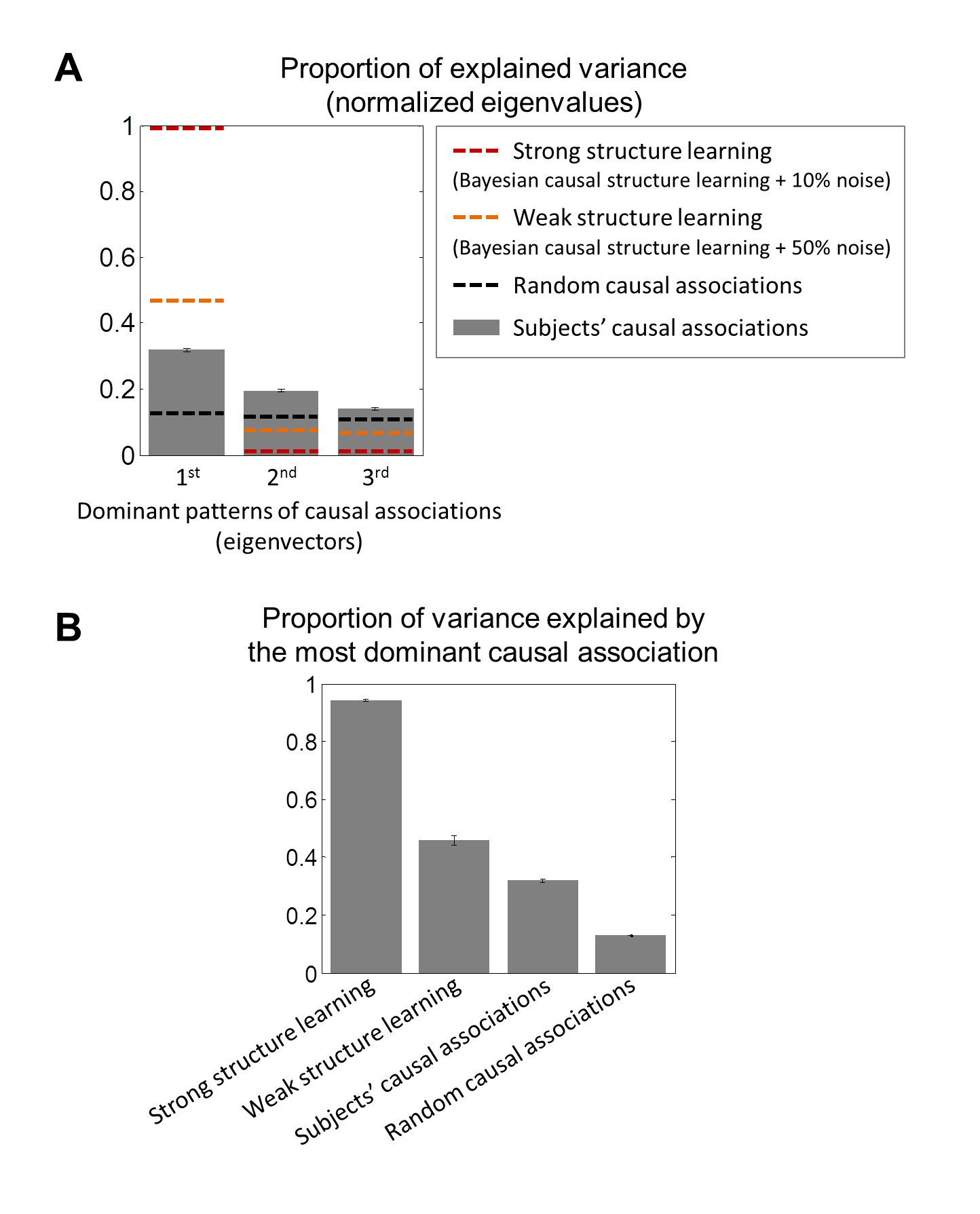

Supplement: S10 Fig — Related to S9 Fig. To quantify statistical regularity in causal associations as would be predicted by a structure-learning effect, we fit the Bayesian causal structure learning model to participants’ causal rating data to infer underlying causal associations and then conducted a principal component analysis on data vectors of the causal associations, followed by quantification of the amount of variance explained by the dominant patterns of causal association. If structure learning is the major factor that affects causal ratings, the dominant patterns of causal association should explain most of the variance. The strong structure learning hypothesis, indicated by the red dotted lines, refers to the case in which it is assumed that the respective learned task structure for the type1 and type2 round is used to make predictions about causal ratings. It is implemented by fitting the Bayesian causal structure learning model to the causal rating data of the type1 and type2 round, respectively, followed by adding 10% noise to its predictions. The weak structure learning hypothesis, indicated by the orange dotted lines, is the same as the strong structure learning one, except for adding 50% noise to its predictions. The subjects’ causal associations, indicated by the grey box plot, refer to the case in which the Bayesian causal structure learning model was fit to each individual round’s data to infer underlying causal associations. The random causal associations, indicated by the black dotted lines, refer to the case in which the causal associations are randomly generated from a uniform distribution. Shown are the proportions of explained variance (A: normalized eigenvalues for the top three eigenvectors; B: for the first eigenvector only) in causal associations predicted by each case. We found that the most dominant patterns of causal associations predicted by the subject’s causal associations model explain only 32% of variance, which is significantly less than the weak str [file pbio.1002137.s014.tif]

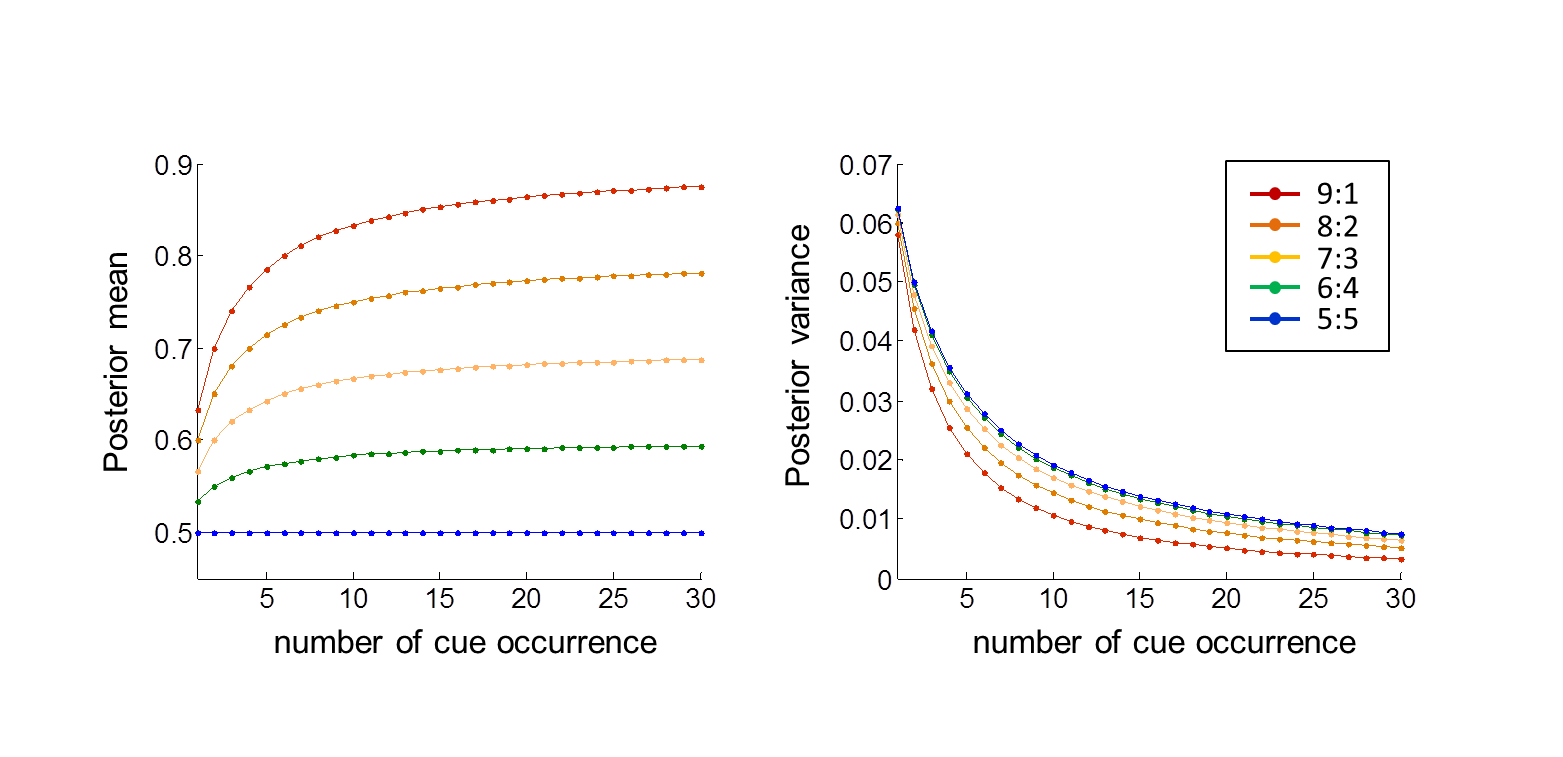

Supplement: S11 Fig — Shown are the posterior mean and variance of the Bayesian causal inference model as a function of the total number of cue occurrence. The model considers two cues and one outcome. The ratio m:n means that the ratio of the occurrence of the first cue and the second cue is m:n. For example, the point of the red line (10,0.83) in the left figure refers to the case in which the first cue was presented nine times out of ten, the second cue was presented one time, and the model’s estimate of the posterior mean was 0.83. There is a rapid update in the early phase of learning and then the update becomes slower as learning progresses, which adequately reflects the modulation of learning rate based on novelty of stimuli [9]. (TIF) [file pbio.1002137.s015.tif]

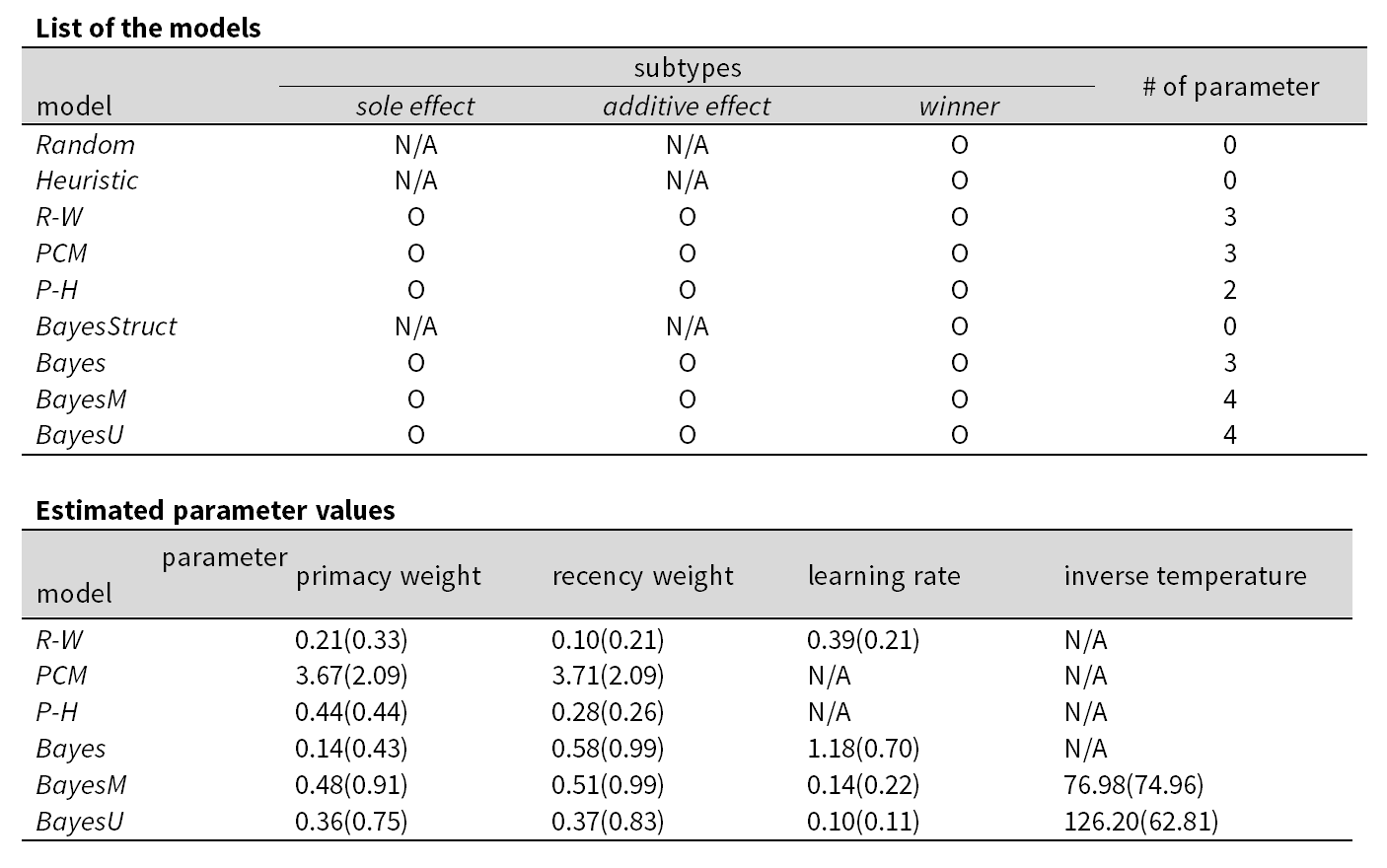

Supplement: S1 Table — List of the models and estimated parameter values. Random: model generating a random causal rating, Heuristic: model of heuristic causal judgment that learns from novel stimulus-novel outcome association, R-W: delta-rule model based on the Rescorla-Wagner model [6], PCM: probabilistic contrast model (Jenkins and Ward, 1965), P-H: Pearce-Hall model [8], BayesStruct: online Monte Carlo Markov Chain method for learning causal Bayesian network structures [48,74], Bayes: Bayesian inference of causal learning, BayesM: a variant of the Bayes model [47] in which the learning rate is determined based on causal strength, BayesU: a variant of the Bayes model [47] in which the learning rate is determined based on causal uncertainty. “O” indicates that the model has been created and tested. “N/A” means the model is not suitable for that type of test. Model subtypes: additive effect refers to the version of models assuming that repetitive presentation strengthens or weakens the likelihood of outcome delivery, and sole effect refers to the version of models assuming that participants take the presence or absence of the stimulus into account when they update the causal strength. Winner refers to which of the model versions was chosen for each participant. A comparison between the sole effect and the additive effect is shown in S5A Fig, and the full model comparison for the winner version is shown in S5B Fig. Note that for model comparison we used leave-one-out cross validation (LOOCV), which provides empirical evaluation of generalization performance of nonprobabilistic learning models [77]. The LOOCV thus takes model complexity into account. Between-subjects mean and standard deviation, 47 subjects; parenthesis: standard deviation. (TIF) [file pbio.1002137.s016.tif]

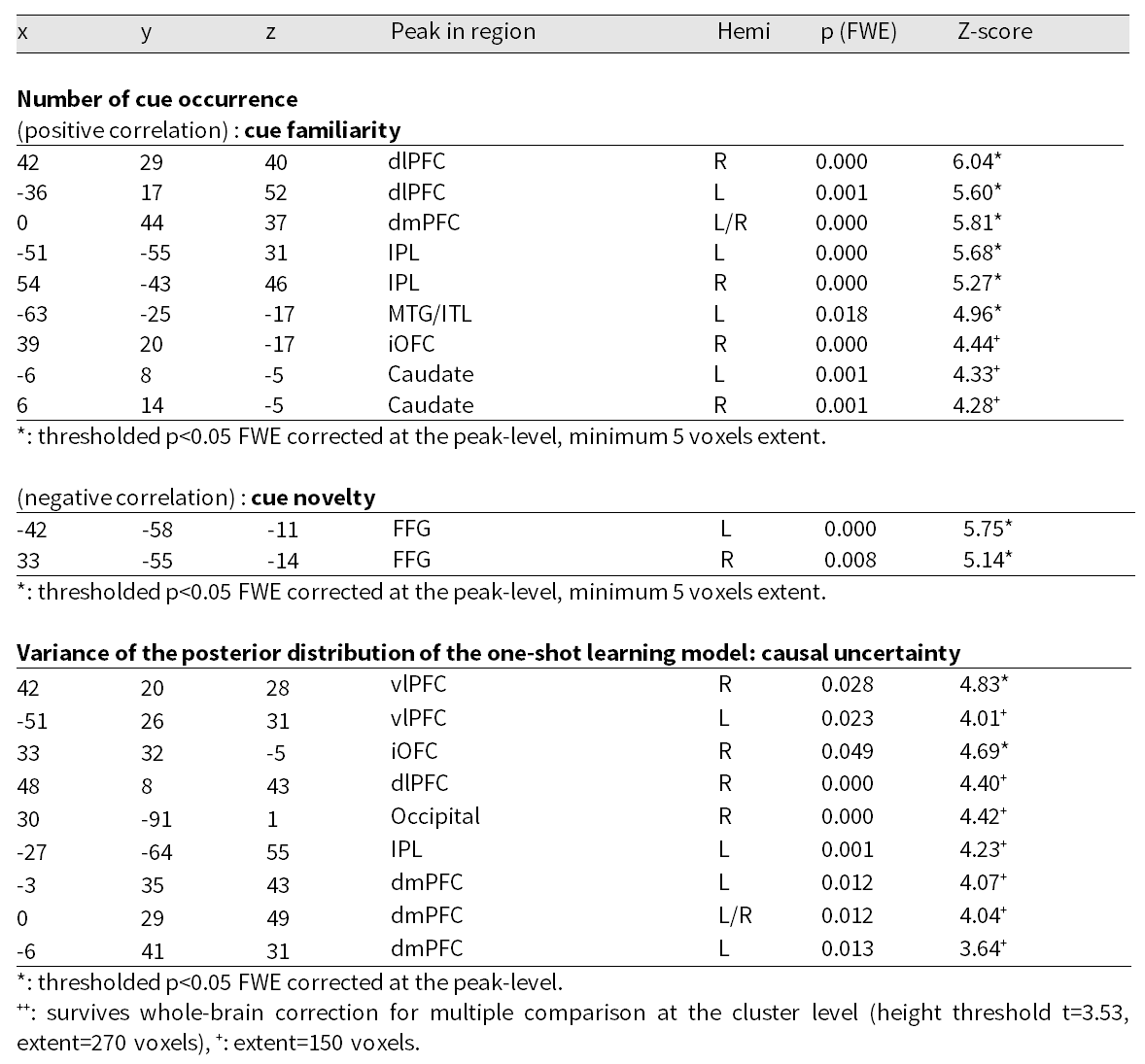

Supplement: S2 Table — p(FWE): corresponds to peak level if the z-score indicates * and corresponds to cluster level if the z-score indicates +. vlPFC, ventrolateral prefrontal cortex; dmPFC, dorsomedial prefrontal cortex; dlPFC, dorsolateral prefrontal cortex; iOFC, inferior orbitofrontal cortex; IPL, inferior parietal lobule; MTG, middle temporal gyrus; ITL, inferior temporal lobe; FFG, fusiform gyrus. All the areas (marked with either “+” or “*”) survived after the-brain correction for multiple comparison at the cluster level (corresponding to “+”; height threshold t = 3.53, extent > 100 voxels). (TIF) [file pbio.1002137.s017.tif]
